# Binary Double Network-like Structure: An Effective Energy-Dissipation System for Strong Tough Hydrogel Design

**DOI:** 10.3390/polym15030724

**Published:** 2023-01-31

**Authors:** Genxin Chen, Sijie Tang, Honghan Yan, Xiongbin Zhu, Huimin Wang, Liya Ma, Kang Mao, Changying Yang, Jiabing Ran

**Affiliations:** 1 College of Biological & Pharmaceutical Sciences, China Three Gorges University, Yichang 443002, China; 2 Hubei Key Laboratory of Natural Products Research and Development, China Three Gorges University, Yichang 443002, China; 3 The Centre of Analysis and Measurement of Wuhan University, Wuhan University, Wuhan 430072, China; 4 State Key Laboratory of Environmental Geochemistry, Institute of Geochemistry, Chinese Academy of Sciences, Guiyang 550081, China

**Keywords:** double-network, hydrogel, toughness

## Abstract

Currently, hydrogels simultaneously featuring high strength, high toughness, superior recoverability, and benign anti-fatigue properties have demonstrated great application potential in broad fields; thus, great efforts have been made by researchers to develop satisfactory hydrogels. Inspired by the double network (DN)-like theory, we previously reported a novel high-strength/high-toughness hydrogel which had two consecutive energy-dissipation systems, namely, the unzipping of coordinate bonds and the dissociation of the crystalline network. However, this structural design greatly damaged its stretchability, toughness recoverability, shape recoverability, and anti-fatigue capability. Thus, we realized that a soft/ductile matrix is indispensable for an advanced strong tough hydrogel. On basis of our previous work, we herein reported a modified energy-dissipation model, namely, a “binary DN-like structure” for strong tough hydrogel design for the first time. This structural model comprises three interpenetrated polymer networks: a covalent/ionic dually crosslinked tightened polymer network (stiff, first order network), a constrictive crystalline polymer network (sub-stiff, second order network), and a ductile/flexible polymer network (soft, third order network). We hypothesized that under low tension, the first order network served as the sacrificing phase through decoordination of ionic crosslinks, while the second order and third order networks together functioned as the elastic matrix phase; under high tension, the second order network worked as the energy dissipation phase (ionic crosslinks have been destroyed at the time), while the third order network played the role of the elastic matrix phase. Owing to the “binary DN-like” structure, the as-prepared hydrogel, in principle, should demonstrate enhanced energy dissipation capability, toughness/shape recoverability, and anti-fatigue/anti-tearing capability. Finally, through a series of characterizations, the unique “binary DN-like” structure was proved to fit well with our initial theoretical assumption. Moreover, compared to other energy-dissipation models, this structural design showed a significant advantage regarding comprehensive properties. Therefore, we think this design philosophy would inspire the development of advanced strong tough hydrogel in the future.

## 1. Introduction

Currently, hydrogels simultaneously featuring high strength, high toughness, superior recoverability, and benign anti-fatigue properties have demonstrated great application potential in a broad array of fields, including biomechanical actuators, biomedical and tissue engineering, ionic skin, soft electronics, soft robotics, etc. [1,2]. In order to develop satisfying hydrogels, great efforts have been made by researchers to elucidate the relationship between the mechanical performance and the composition/structure/topology of different hydrogels [3,4]. Intuitively, the strength and anti-fatigue property of a hydrogel depend on its capability to resist mechanical stress or cyclic mechanical stress; its toughness is determined by the synergistic effect of its strength and stretchability; and its recoverability results from its elasticity [4]. Structurally speaking, the strength of a hydrogel is positively related to the number of hidden chains within the hydrogel network. In this regard, constructing molecular entanglements [5] and molecular crystallites [6] or increasing polymer fraction [7] and cross-linking density [4] has proven to be capable of increasing the number of hidden chains, therefore enhancing the strength of a hydrogel. The toughness of a hydrogel commonly arises from the following three factors: (i) scission of a layer of polymer chains on the crack tip (intrinsic contribution); (ii) hysteric mechanical dissipation in the bulk materials around the crack tip due to the Mullins effect and viscoelasticity. For instance, non-covalent cross-links [8] and slide-ring crosslinks [9] are representative structural moieties which could substantially dissipate accumulated stress within the hydrogel network; (iii) near-crack dissipation owing to the dissociation of polymer entanglements or crystallites [5]. The recoverability of a stretched hydrogel is mainly influenced by the rearrangement capability of the polymer chains. Constructing reversible crosslinks [10] or flexible/ductile matrix network [11], to some extent, could aid an extended hydrogel recover to its initial shape, size, and mechanical properties. The anti-fatigue capability of a hydrogel is mainly affected by the ability of the polymer network to resist crack propagation. Zhao et al. thought that the anti-crack capability of a hydrogel was highly related to the fraction of polymer crystalline domain [12] and the extent of anisotropic orientation of polymer chains [13] within the hydrogel.

**Figure 1:**
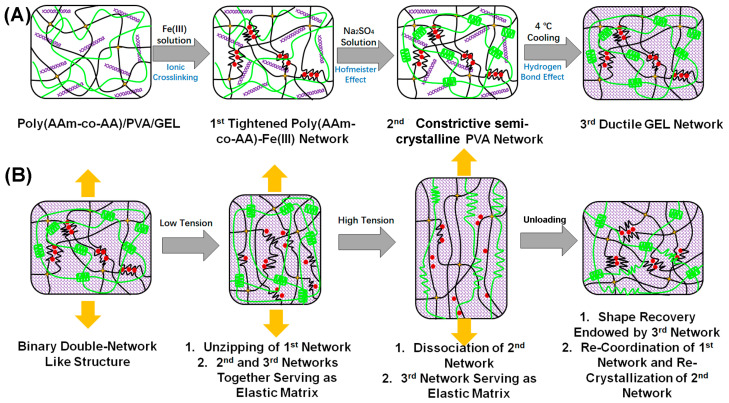
Schematic diagrams of (**A**) the preparation process of the “binary DN-like” hydrogel and (**B**) the rationale behind the energy dissipation and shape recovery of the “binary DN-like” hydrogel subjected loading-unloading treatment.

DN structure is a type of classical energy-dissipation model for strong tough hydrogel design, the rationale behind which can be summarized as follows: a highly cross-linked tightened/stiff network substantially depletes accumulated energy through the breakage of covalent bonds under hydrogel deformation, whilst a loosely cross-linked soft/ductile network plays the role of elastic matrix to make the hydrogel stretchable and self-recoverable [3]. Fractured covalent bonds can hardly be restored, so a typical DN hydrogel commonly exhibits inferior toughness recoverability and anti-fatigue capability [14]. In this regard, many DN-like models which utilize the breakage of reversible bonds to dissipate energy have been developed to solve the above-mentioned problems [15,16,17]. Inspired by the DN-like structure, in our previous work, we reported ahydrogel, which had two consecutive energy-dissipation systems, namely, the unzipping of coordinate bond ions and the disassembly of the semi-crystalline network. The as-prepared hydrogel demonstrated high strength and high toughness, but its stretchability, toughness recoverability, shape recoverability, and anti-fatigue capability were far from satisfactory [1]. The reason behind this phenomenon, we inferred, arose from the lack of a ductile/soft network, on the basis of which, we proposed a modified design philosophy termed the “binary DN-like” structure for synthesizing advanced strong tough hydrogels (Figure 1A). In this scheme, a hydrogel comprises three independent but synergistic structural moieties: a covalent/ionic dually cross-linked tightened polymer network (first network, stiff), a constrictive semi-crystalline polymer network (second network, sub-stiff), and a flexible and ductile matrix network (third network, soft). The three networks are interpenetrated, forming a special “binary DN-like” structure: (i) under low tension, the first network serves as the main sacrificing phase to consume energy via the unzipping of ionic crosslinks, while the second and third network together function as the elastic matrix to maintain recoverability; (ii) with the increase of tension, ionic crosslinks are gradually destroyed, and the second network becomes the main sacrificing phase to dissipate energy through the disassembly of the semi-crystalline polymer network, while the third network plays the role of the elastic phase to maintain high recoverability of the hydrogel (Figure 1B). Theoretically, the as-prepared hydrogel should have two successive energy-dissipation systems, so it was assumed to have extremely high toughness. In addition, owing to the presence of the elastic matrix phase under both low tension and high tension, the “binary DN-like” structured hydrogel should have excellent shape and size recoverability. Because both coordinate bond and hydrogen bond are dynamically reversible, the “binary DN-like” hydrogel, in principle, should demonstrate excellent toughness recoverability. Owing to the presence of semi-crystalline domains, the as-prepared hydrogel could resist crack propagation, thereby having an outstanding anti-fatigue property.

In this work, a N,N′-methylenebisacrylamide (MBAA)/Fe(III) dually crosslinked poly(acrylamide-co-acrylic acid) (poly(AAm-co-AA) network was utilized as the first network (Figure S1, Supplementary Materials) [16], which has proven to have an isotropic energy-dissipation manner [18]. Hofmeister effect induced semi-crystalline polyvinyl alcohol (PVA) network was utilized to form the second network [1], and cooling induced gelatin (GEL) network was applied to construct the third network [19]. Herein, we called the as-prepared hydrogel as poly(AAm-co-AA)/PVA_x_/GEL_y_-Fe(III). Subscript x and y meant the initial feeding ratio of PVA and GEL, respectively. Poly(AAm-co-AA)/PVA/GEL-Fe(III), whose subscripts were not designated, referred to this kind of hydrogel ignoring its feeding composition. We firstly testified the rationality of the “binary DN-like” structure through elaborate contrast experiments. Then, we systematically investigated the tensile stress–strain behaviors, energy-dissipation behaviors, toughness/shape recovery behaviors, and anti-fatigue behaviors of as-prepared hydrogel. We hoped this kind of structural design philosophy could enlighten advanced strong tough hydrogel fabrication in the future.

## 2. Experimental Section

### 2.1. Materials

Acrylamide (AAm, 99%) were purchased from Aladdin Biochemical Technology Co., Ltd. (Shanghai, China). N,N,N′,N′-Tetramethylethylenediamine (TEMED, ≥98%) was purchased from Kemiou Chemical Reagent Co., Ltd. (Tianjin, China). Benzoin dimethyl ether (DMPA, 99%) was purchased from Shanghai Macklin Biochemical Co., Ltd. (Shanghai, China). Sodium sulfate (Na_2_SO_4_, ≥99%) was bought from Chengdu Kelong Chemical Co., Ltd. (Chengdu, China). N,N′-Methylenebisacrylamide (MBAA, ≥98%), Iron (III) chloride hexahydrate (FeCl_3_·6H_2_O, ≥99%), Acrylic acid (AA, ≥99%), Polydimethylsiloxane (PDMS) and Polyvinyl alcohol (1750 ± 50, ≥99%) were purchased from Sinopharm Chemical Reagent Co., Ltd. (Shanghai, China). Gelatin was bought from Sigma-Aldrich Co. Ltd. (Shanghai, China). All the reagents were used as received without purification. dd water was used throughout the whole experiment.

### 2.2. Synthesis of Hydrogels with Different Initial Feeding Composition

In a typical protocol, a certain quality of PVA was firstly dissolved in 10 mL dd water at 95 °C for 30 min, and then cooled down to 45 °C. After that, a certain quality of gelation was added into the above solution, and stirred for 30 min until it was completely dissolved. Next, AAm and AA were added into the above solution with agitation for 15 min, followed by adding 118 μL aqueous solution of MBAA (10 mg/mL), 100 μL ethanol solution of DMPA (50 mg/mL) and 8 μL TEMED in sequence. Next, the solution was degassed with an ultrasonic device at 40 KHz for 5 min (Scientz, Ningbo, China), dropped into a Teflon mould (dumbbell-shape mould for tensile test and rectangle mould for tearing test), and then subjected to UV irradiation at 254 nm for 1 h (Scientz, Ningbo, China). Afterwards, the resultant samples were placed in a sealed PE bag for 12 h, followed by soaking them in 0.1 M FeCl_3_ solution for 15 min. The as-obtained samples were subsequently placed in a sealed PE bag for 12 more hours. Then, they were soaked in 1.8 M Na_2_SO_4_ solution for 20 min and placed in a sealed PE bag for 12 h. Finally, the as-prepared hydrogels were surface smeared with silicon oil to prevent evaporation of water and then placed in 4 °C refrigerator for 24 h. The specific feeding compositions of different groups are listed in Table S1 (Supplementary Materials).

### 2.3. Characterizations

X-ray Diffraction (XRD): XRD analysis (SmartLab, Tokyo, Japan) was utilized to investigate the crystal phases of these hydrogels. The working condition of XRD was CuK_0_ radiation via a rotating anode at 40 kV and 40 mA. The data were collected in a step of 0.1° and a range of diffraction angles (2θ) from 5° to 60°. Each peak was separated peakfit software (Peakfit 4.12, Systat Software GmbH, San Jose, CA, USA).

Scanning Electron Microscope (SEM): The as-prepared hydrogels were firstly placed in a refrigerator at −20 °C for 24 h and then lyophilized at −60 °C for 3 days. The lyophilized samples were sputtered with gold for 120 s and then observed by SEM (Toshiba, Tokyo, Japan).

Mechanical Properties Test: All the tests were performed in air (25 °C, 45% humidity) using a Universal Testing Machine (QingJi, Shanghai, China). All the force–displacement profiles were transformed into nominal stress–strain curves.

Tensile Test: These samples were maintained in a dumbbell-shape (60 mm × 5 mm × 1.5 mm). The characteristic length (l_0_) was controlled at 30 mm. The tensile rate was maintained at 50 mm/min. The stress (*σ*) was obtained by dividing the force (*F*) by the cross-sectional area, and the strain (*ε*) was obtained by dividing the stroke (*l_t_*) by l_0_. The Young’s modulus (*E*) was calculated from the slope of the initial part (strain < 10%) of the tensile stress–strain curves. The fracture stress and fracture strain were directly given by the machine after each test. The fracture energy (*W*) was calculated by area below the stress–strain curve of a sample, multiplying its characteristic length l_0_.

(1) $$\sigma =\frac{F}{w\;\times \;t}\;$$

(2) $$\epsilon =\frac{l_{t}}{l_{0}}\;$$

(3) $$E=\frac{\sigma_{A}-\sigma_{B}}{\varepsilon_{A\;}-\varepsilon_{B}}$$

(4) $$W=l_{0}\int_{0}^{\varepsilon_{f}}\sigma d_{\varepsilon }$$

where *F*, *E*, *ε*, σ, and *W* refer to force, Young’s modulus, strain, stress, strain, and fracture energy, respectively. l_0_, *l_t_*, *w*, and *t* are characteristic length, displacement at time of t, width, and thickness of a targeted specimen. *σ_A_*, *σ_B_*, *ε_A_*, and *ε_B_* represent stress at *A*, stress at *B*, strain at *A*, and strain at *B*, respectively. *A* and *B* are corresponded to two points on the stress–strain curve with strain <10%. *ε_f_* is the fracture strain. Each value was the mean for three replicates.

Tearing Test: The gels were in trousers-shape (90 mm × 20 mm × 2 mm) with an initial notch of 20 mm. The two arms were clamped and extended at 100 mm/min. Three replicates were carried out for each group. The tearing energy (*T*) amounts to the work required to tear a unit area. The work done during a tear test can be calculated by

(5) $$\Delta W=2F_{ave}\Delta c$$

where *F_ave_* refers to the average force of peak values during steady-state tear and Δc is the tear distance.

The tearing energy *T* can be expressed as

(6) $$T=\frac{\Delta W}{B\Delta c}$$

where B means the thickness of specimen. Thus, through combining Equations (5) and (6)

(7) $$T=\frac{2F_{ave}}{B}\;$$

Cyclic Loading–Unloading Test: Nominal tensile samples were extended with a maximum extension ratio of 1.5, 2, 2.5, 3, 3.5, 4, 4.5, 5, 5.5, 6, 6.5, 7, 7.5, 8.0, 8.5, and 9 (denoted as λ_max_ = 1.5, 2, 2.5, 3, 3.5, 4, 4.5, 5, 5.5, 6, 6.5, 7, 7.5, 8.0, 8.5, or 9) and then unloaded. Each group was tested 3 times. The tensile rate was maintained at 100 mm/min. The dissipated energy (*U_hys_*) was estimated by area between the loading–unloading curves:

(8) $$\lambda_{\max}=\frac{l_{t}}{l_{0}}\;$$

(9) $$U_{hys}=\int_{0}^{\varepsilon_{f}}\left(\sigma_{loading}-\sigma_{unloading}\right)d_{\varepsilon }$$

where *λ_max_* and *U_hys_* refer to maximum extension ratio and dissipated energy, respectively. *σ_loading_* and *σ_unloading_* refer to stress under loading and unloading processes, respectively.

Successive Loading–Unloading Test (No Resting Time): The loading–unloading tests were repeatedly carried out on the same sample, with increasing λ_max_ (1.5, 2, 2.5, 3, 3.5, 4, 4.5, 5, 5.5, 6, 6.5, 7, 7.5, 8.0, 8.5, 9, 10, and 10.5), until the sample was finally fractured. No resting time was given between any two successive loadings. The tensile rate was maintained at 100 mm/min. Each group was tested for 3 times.

Toughness Recovery Test (Different Resting Time): A nominal tensile sample was extended with a preset maximum extension ratio (3, 5, or 7) and then unloaded. After being recovered for a period of time, a same loading–unloading test was conducted on the sample. Toughness recovery percentage was evaluated by the ratio of dissipated energy after different recovery time divided by that of the first loading–unloading test. The tensile rate of loading–unloading tests was maintained at 50 mm/min. Each value was the mean for three replicates.

Shape Recovery Assay: A nominal specimen was subjected to a single loading–unloading test with a preset maximum extension ratio (3, 5, or 7). The time a specimen took for recovering to its original shape and size (visual observation) was recorded with a timer. Each value was the mean for three replicates.

Fatigue-Resistance Test (Fixed Resting Time): A nominal tensile sample was extended with a preset maximum extension ratio (3, 5, or 7) and then unloaded. After resting for 0, 5, 10, or 15 min, the same loading–unloading test was carried out on the sample. The operations were repeatedly conducted for 5 times. The toughness retention percentage was calculated by the ratio of dissipated energy after each tension divided by that of the first loading–unloading test. The tensile rate of loading–unloading tests was maintained at 100 mm/min. Each value was the mean for three replicates.

Statistical Analysis: Statistical analysis was carried out by one-way analysis of variance (ANOVA) for post hoc comparison using GraphPad Prism 7 (GraphPad Software Inc., San Diego, CA, USA). *p* < 0.05 was considered statistically significant.

## 3. Results and Discussions

### 3.1. Identification of the “Binary DN-like” Structure of the As-Fabricated Poly(AAm-co-AA)/PVA/GEL-Fe(III) Hydrogel

**Figure 2:**
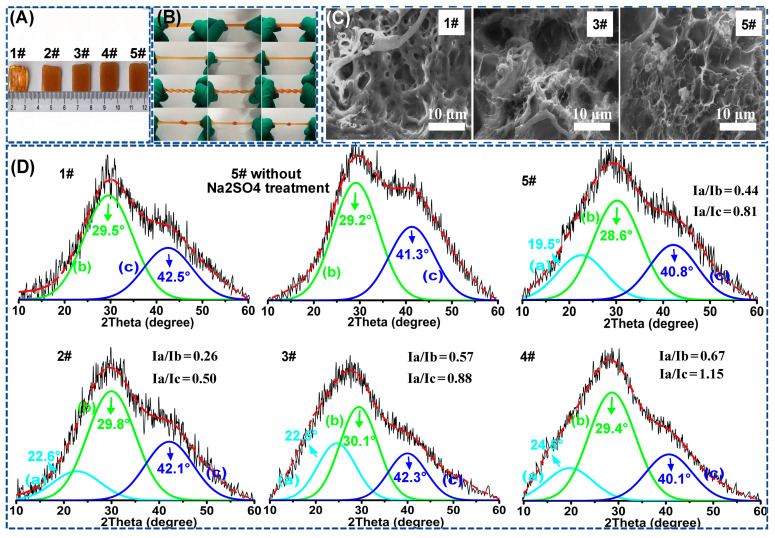
Visual demonstration of the (**A**) mechanical performances including stretching, twisting, and knotting and (**B**) transparency of sample 1#–5#. (**C**) SEM images of sample 1#, 3#, and 5 #; (**D**) XRD spectrums of sample 1#, 2#, 3#, 4#, 5#, and poly(AAm-co-AA)/PVA_0.3_-Fe(III) (5#) without Na_2_SO_4_ treatment. Each peak was separated using Peakfit. I_a_/I_b_ and I_a_/I_c_ refer to the intensity ratios of peak a/peak b and peak a/peak c, respectively.

In order to testify the rationality of the strategy, both the macroscopically physical-chemical properties and the microscopically structural and topological composition of the as-fabricated hydrogels were investigated in detail. In Figure 2A, it could be found that the as-fabricated five hydrogels (1# poly(AAm-co-AA)/GEL_0.3_-Fe(III); 2# poly(AAm-co-AA)/GEL_0.2_/PVA_0.1_-Fe(III), 3# poly(AAm-co-AA)/GEL_0.15_/PVA_0.15_-Fe(III), 4# poly(AAm-co-AA)/GEL_0.1_/PVA_0.2_-Fe(III), and 5# poly(AAm-co-AA)/PVA_0.3_-Fe(III)) had roughly similar shape and size. In addition, we also calculated the water contents of samples 1#–5#, which were 60.37%, 58.03%, 58.71%, 60.12%, and 59.99%, respectively (Figure S2, Supplementary Materials). Obviously, the water contents of the five samples were roughly identical, either. In the following experiment, maintaining identical shape, size, and water content of these hydrogels were indispensable for investigating the “binary DN-like” structural model because the three factors might have huge influence on the mechanical properties of the hydrogel.

As shown in Figure 2B, it can be observed that the as-prepared poly(AAm-co-AA)/GEL/PVA-Fe(III) hydrogel had superior structural flexibility because it could be stretched, twisted, and knotted. Owing to the incorporation of Fe(III), all five specimens demonstrated a homogeneous brownish red (Figure 2B), from which it could be concluded that a large number of Fe(III) were homogeneously dispersed in the hydrogel matrix, and they, in principle, were sufficient to induce the formation of the tightened poly(AAm-co-AA)-Fe(III) network through Fe(III)-COO^–^ coordination. Additionally, we also found that sample 1# was totally transparent, while sample 5# was totally non-transparent. In addition, the transparency of the five samples was decreased as follows: 1# > 2# > 3# > 4# > 5#. Herein, the transparency is negatively correlated to the content of PVA semi-crystalline domains within the poly(AAm-co-AA)/GEL/PVA-Fe(III) hydrogel. The lower the transparency, the higher the density of the semi-crystalline PVA network. Through the above analysis (Figure 2B), we could conclude that the Hofmeister effect induced the formation of a semi-crystalline PVA network within the poly(AAm-co-AA)/PVA/GEL-Fe(III) hydrogel. Moreover, from sample 2# to 5#, the density of the PVA semi-crystalline network was gradually increased. From the surface morphology observation (Figure 2C), it could also be found that the poly(AAm-co-AA)-Fe(III) network, PVA network, and GEL network were interpenetrated and no obvious phase separation could be observed within the poly(AAm-co-AA)/GEL/PVA-Fe(III) hydrogel. In addition, both sample 1# and sample 5# took on an obvious porous structure, while sample 3# exhibited a densified and nonporous structure, which meant that less structural defects were generated within the poly(AAm-co-AA)/GEL/PVA-Fe(III) hydrogel. Figure 2D shows the XRD spectra of sample 1#, 2#, 3#, 4#, 5# and poly(AAm-co-AA)/PVA_0.3_-Fe(III) (5#) without Na_2_SO_4_ treatment. Here, each peak was separated, and the peaks at around 30° and 41° corresponded to the diffraction patterns of the poly(AAm-co-AA)-Fe(III) network, while the peak at around 22° corresponded to the semi-crystalline PVA network. As for the different sample, these peaks slightly shifted, which could be ascribed to the intramolecular interaction between PVA and either GEL or poly(AAm-co-AA). In addition, we also calculated the relative variation of intensity of the diffraction peaks of these samples (i.e., peak 22°/peak 30° and peak 22°/peak 41°, corresponding to peak a/peak b and peak a/peak c in Figure 2D, respectively). It could be found that with the increase of initial PVA feeding amount, the density of the semi-crystalline PVA network within the poly(AAm-co-AA)/GEL/PVA-Fe(III) hydrogel was gradually increased (Figure 2D), which was in agreement with the results of the transparency observation (Figure 2A).

**Figure 3:**
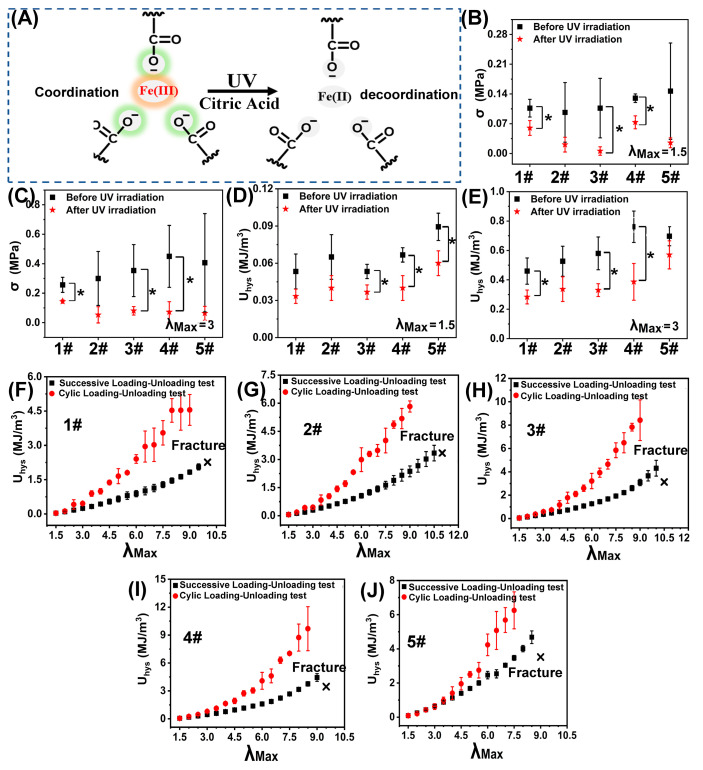
(**A**) Schematic diagram showing the decoordination of Fe(III)-COOO^−^ under UV radiation; Tensile stress and dissipation energy of sample 1#, 2#, 3#, 4#, and 5 # under low tension of λ_max_ = 1.5 (**B**,**D**) and λ_max_ = 3 (**C**,**E**) before and after UV radiation (* *p* < 0.05); Dissipation energy of sample 1# (**F**), 2# (**G**), 3# (**H**), 4# (**I**), and 5 # (**J**) subjected to cyclic loading–unloading test and successive loading–unloading test.

We assumed that under low tension, the poly(AAm-co-AA)-Fe(III) network was highly tightened (stiff) and the unzipping of Fe(III)-COO^−^ coordination was the main energy dissipator, while the constrictive semi-crystalline PVA network (sub-stiff) and the ductile GEL network (soft) together served as the elastic matrix phase to maintain the recoverability of the hydrogel. Under high tension, the Fe(III)-COO^−^ coordination was destroyed and the semi-crystalline PVA network became the main contributor of energy dissipation through the disassembly of the PVA semi-crystalline domains, while the flexible GEL network played the role of the elastic matrix to maintain the recoverability of the hydrogel. Herein, cyclic loading–unloading tests (λ_max_ = 1.5 or 3) were carried out on the five specimens (1#–5#). Simultaneously, the same operation was performed on five identical samples that had been surface-sprayed with 0.2 M citric acid solution and subjected to UV radiation for 10 min. By doing this, the Fe(III) was reduced to Fe(II) [20], and the coordination between Fe(III) and COO^–^ would be undermined (Figure 3A). It could be found that once the Fe(III)-COO^–^ coordination structure was destroyed, the stress and U*_hys_* were extensively descended (Figure 3B–E). For each group, if λ_max_ < 3 (low tension), the U*_hys_* value in a cyclic loading–unloading test was the same with that in a successive loading–unloading test. In addition, the U_hys_ values under low tension (λ_max_ = 1.5 or 3) for different groups are nearly identical (Figure 3F–J). This indicated that under low tension, the topological structure of either the GEL network or the PVA network was not destroyed, they, therefore, were not the main contributors for energy dissipation. The energy dissipation could only arise from the unzipping of Fe(III)-COO^−^. Thus, this phenomenon, to some extent, confirmed that under low tension, the unzipping of Fe(III)-COO^−^ was the main contributor for energy dissipation. In conclusion, under low tension, the poly(AAm-co-AA)-Fe(III) served as the sacrificing phase to dissipate energy, while the GEL network and the PVA network together functioned as the elastic matrix, forming the first order DN-like structure.

**Figure 4:**
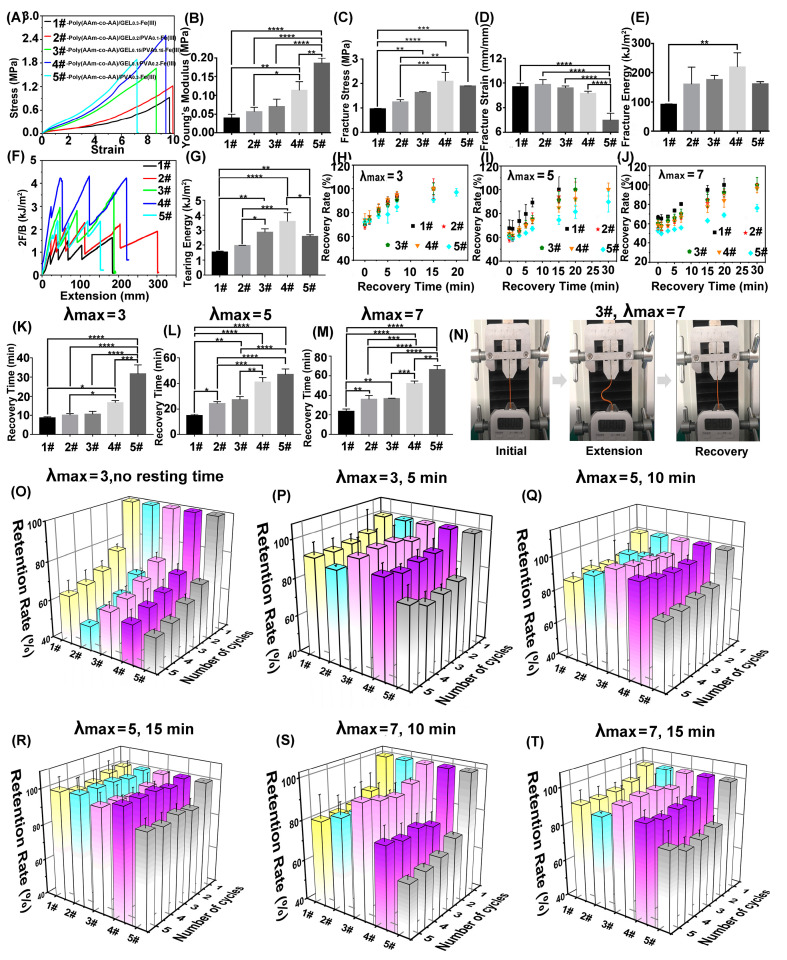
(**A**) Representative stress–strain curves of poly(AAm-co-AA)/GEL_0.3_-Fe(III) (1#), poly(AAm-co-AA)/GEL_0.2_/PVA_0.1_-Fe(III) (2#), poly(AAm-co-AA)/GEL_0.15_/PVA_0.15_-Fe(III) (3#), poly(AAm-co-AA)/GEL_0.1_/PVA_0.2_-Fe(III) (4#), and poly(AAm-co-AA)/PVA_0.3_-Fe(III) (5#), and histograms showing the corresponding average values of (**B**) Young’s modulus, (**C**) fracture stress, (**D**) fracture strain, and (**E**) fracture energy; Representative force-extension curves (**F**) and histograms (**G**) showing the average tearing energy of sample 1#, 2#, 3#, 4#, and 5# subjected to tearing test; Tough recovery properties of the five hydrogels extended at λ_max_ of 3 (**H**), 5 (**I**), and 7 (**J**) and then given a resting time of 0, 1, 3, 5 7, 15, and 30 min, respectively; Shape recovery time of the five hydrogels extended at λ_max_ of 3 (**K**), 5 (**L**), and 7 (**M**). (**N**) shows the shape recovery process of sample 3# extended at a λ_max_ of 5; Histograms showing the toughness retention rate of the five hydrogels in five successive loading–unloading tests at different λ_max_ and different resting time: (**O**) λ_max_ = 3, no resting time; (**P**) λ_max_ = 3, resting time = 5 min; (**Q**) λ_max_ = 5, resting time = 10 min; (**R**) λ_max_ = 5, resting time = 15 min; (**S**) λ_max_ = 7, resting time = 10 min; and (**T**) λ_max_ = 7, resting time = 15 min. * *p* < 0.05, ** *p* < 0.01, *** *p* < 0.005, and **** *p* < 0.001.

To further verify the “binary DN-like” structure, cyclic/successive loading–unloading tests under high tension (λ_max_ > 3) were carried out on sample 1#–5# as well. When the λ_max_ surpassed 3, it could be found that for each group, the U*_hys_* value in a cyclic loading–unloading test was much higher than that in a successive loading-unloading test, which meant that the Fe(III)-coordination had been destroyed. At a given λ_max_ (λ_max_ > 3), the U*_hys_* values were decreased as following sequence: 4# > 3# > 5# > 2# > 1# (Figure 3F–J). Obviously, under high tension, no matter the GEL network (1#) or the PVA network (5#) or the PVA/GEL (2#–4#) network, their original topological structure was gradually destroyed, and all of them could dissipate energy. In addition, the energy dissipation capability of the semi-crystalline PVA network was much higher than the soft GEL network because much more energy was needed to undermine the semi-crystalline PVA network than to undermine the non-crystalline GEL network. Interestingly, we also observed that the interpenetrated GEL/PVA network demonstrated higher energy dissipation capability than the single GEL network or single PVA network, which could be explained by the near-crack dissipation theory proposed by Zhao et al. [5]. Besides, we further compared the shape recovery time of the five samples under high tension (Figure 4K–M). Obviously, the GEL network showed a much faster shape recovery property than the PVA network under high tension, which meant that the elasticity of the GEL network was much higher than the PVA network. Thus, it could be concluded that under high tension, the semi-crystalline PVA network became the main contributor of energy dissipation, while the flexible GEL network played the role of the elastic matrix, forming the second order DN-like structure. In summary, the assumption about the “binary DN-like” structure of the poly(AAm-co-AA)/GEL/PVA-Fe(III) hydrogel was confirmed by the experimental data.

### 3.2. Mechanical Properties of the “Binary DN-like” Hydrogel

**Figure 5:**
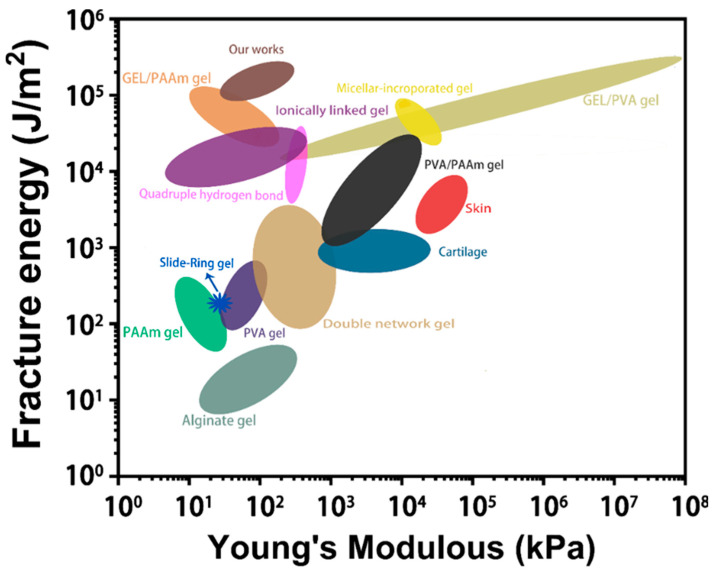
Comparisons of toughness of diverse structural models for dissipating energy [21,22,23,24,25].

To evaluate the mechanical properties of the “binary DN-like” hydrogel, we firstly investigated its tensile stress–strain behaviors (Figure 4A). Generally, the poly(AAm-co-AA)/GEL_0.3_-Fe(III) hydrogel (1#) featured high stretchability, low Young’s modulus, and low strength, owing to the high flexibility of the GEL network (Figure 4B–E). In contrast, the poly(AAm-co-AA)/PVA_0.3_-Fe(III) hydrogel (5#) featured high strength, high stiffness, and low stretchability due to the inflexibility of the semi-crystalline PVA network (Figure 4B–E). Through constructing the “binary DN-like” structure, the stretchability of the poly(AAm-co-AA)/GEL/PVA-Fe(III) hydrogel compared to sample 5# was greatly enhanced and close to that of sample 1# (Figure 4B,D). For instance, the fracture strain of sample 2# was almost approximated to that of sample 1# but was 41.8% higher than that of sample 5#. More importantly, the poly(AAm-co-AA)/GEL/PVA-Fe(III) hydrogel also demonstrated significant advantages in strength and toughness over sample 1# and sample 5#. For instance, the fracture stress of sample 4# was 116.7% higher than that of sample 1# and 10.1% higher than that of sample 5#. Its facture energy, namely toughness, was 138.2% higher than that of sample 1# and 35.2% higher than that of sample 5#, and reached up to record high value of 0.22 MJ/m^2^. In Figure 5, we compared the energy dissipation capability of diverse available structural models and found that the “binary DN-like” structure had great superiority.

Owing to the presence of crystalline domains within the “binary DN-like” structure, the poly(AAM-co-AA)/GEL/PVA-Fe(III) hydrogel also exhibited stronger anti-tearing capability than sample 1# and sample 5# (Figure 4F). For example, the tearing energy of sample 4# was around 3589.21 J/m^2^, which is 128.2% higher and 38.6% higher than that of sample 1# and sample 5#, respectively (Figure 4G). In order to highlight the toughness recovery capability of the “binary DN-like” hydrogel, we calculated the time-dependent dissipation energy recovery percentages of the five samples at λ_max_ of 3, 5, and 7, respectively (Figure 4H–J). Compared to sample 1# and sample 5#, the poly(AAm-co-AA)/GEL/PVA-Fe(III) hydrogel (i.e., sample 2#–4#) showed significantly weaker toughness recovery capability than the former but obviously stronger toughness recovery capability than the latter. At λ_max_ of 7, the toughness of sample 2#–4# could be completely recovered within 30 min, while sample 5# could only restore 76% of its initial energy dissipation capability. However, as for sample 1#, its toughness could be totally recovered within 20 min. Therefore, it could be concluded that the enhanced toughness recovery capability of the poly(AAm-co-AA)/GEL/PVA-Fe(III) hydrogel compared to sample 5# could be attributed to the construction of a “binary DN-like” structure, especially the high elasticity of the GEL network (Figure 4J). In Table S8 (Supplementary Materials), we compared the toughness recovery capability of diverse structural models for energy dissipation. Obviously, the “binary DN-like” structure demonstrated a huge advantage in toughness recovery properties over other structural models, especially at high tension. From the shape recovery time assay (Figure 4K–M and Table S8 (Supplementary Materials)), it could be found that owing to the higher flexibility and softness of GEL compared to semi-crystalline PVA, a stretched sample 1# exhibited much shorter shape recovery time than a stretched sample 5#. Regarding the “binary DN-like” structured poly(AAm-co-AA)/GEL/PVA-Fe(III) hydrogel, its shape recovery time was in the range between that of sample 1# and sample 5#. Through finding the superior feeding ratio of PVA and GEL, the shape recovery time of the poly(AAm-co-AA)/GEL/PVA-Fe(III) hydrogel could be further optimized to extremely approaching that of sample 1#. In Supplementary Videos S1–S15 (Supplementary Materials), the shape recovery behaviors of the five samples (λ_max_ = 5) was demonstrated, from which, we could find better shape recovery capability of “the binary DN-like” structured hydrogel. When being stretched to λ_max_ = 5, sample 3# could be fully recovered to its initial state within 25 min (Figure 4N). Moreover, we also tested and calculated the toughness values of these hydrogels, which had been stretched but were given enough time to recover to their original shape, and found that the as-obtained data were even higher than their initial toughness (Figures S15–S19 (Supplementary Materials)). Owing to the fact that these hydrogels were surface-sealed with silicon oil, the influence of dehydration on their toughness could be eliminated. Obviously, after a loading–unloading test (high tension), no matter the GEL network or the PVA network or their composite network, they could not be completely recovered to their original topological structure, though their shape seemed to be completely recovered; however, the unidirectional stretch helps improve the hydrogen interaction within the GEL network or the PVA network.

In Figure 4O–T, the anti-fatigue capability of the “binary DN-like” structure was investigated in detail through calculating the toughness retention rate of these hydrogels in five consecutive loading–unloading tests. If no resting time was given between two successive loading–unloading tests, all the groups demonstrated an inferior toughness retention rate (Figure 4O). However, if a certain time of resting was given between two consecutive loading–unloading tests, the “binary DN-like” structure demonstrated a significant advantage in anti-fatigue capability over the single DN-like structure. For instance, sample 3# could retain 100% of its initial toughness at λ_max_ of 5 (resting time of 5 min), while the corresponding data for sample 1# and sample 5# were 80% and 89%, respectively (Figure 4Q). At λ_max_ of 7, sample 3# could restore 97% of its initial toughness (resting time of 10 min), while sample 1# and sample 5# could only retain 72% and 62% of their initial toughness, respectively (Figure 4S).

## 4. Conclusions

Strong tough hydrogels have found applications in diverse fields, so developing an advanced structural model for strong tough hydrogel design is of great significance. Inspired by the DN-like theory, we, in our previous work, developed a novel high-strength/high-toughness poly(AAm-co-AA)/PVA-Fe(III) hydrogel, which had two consecutive energy-dissipation systems, namely, the unzipping of Fe(III)-COO^–^ coordination and the dissociation of the PVA crystalline network. However, this structural design greatly damaged its stretchability, toughness recoverability, shape recoverability, and anti-fatigue capability, from which, we realized that a soft/ductile matrix is indispensable for an advanced strong tough hydrogel. In this regard, we, in this work, proposed a modified “binary DN-like” structure for advanced strong tough hydrogel design. This structure comprises a covalent/ionic crosslinked tightened polymer network (stiff, first order network), a constrictive semi-crystalline polymer network (sub-stiff, second order network), and a ductile/flexible polymer network (soft, third order network). We hypothesized that under low tension, the first order network served as the sacrificing phase to dissipate energy through the decoordination of ionic crosslinks, while the second order network and third order network together functioned as the elastic matrix phase; under high tension, the second order network worked as the energy dissipation phase, while the third order network played the role of the elastic phase. Through a series of characterizations, the specific “binary DN-like” structure was proved to fit well with our initial theoretical assumption. In addition, a “binary DN-like” hydrogel, compared to other structured hydrogels, demonstrated better energy dissipation capability, toughness/shape recoverability, and anti-fatigue/anti-tearing capability. The fracture energy of sample 4# (poly(AAm-co-AA)/GEL_0.1_/PVA_0.2_-Fe(III)) with a “binary DN-like” structure reached up to a record-high value of 0.22 MJ/m^2^. Its tearing energy was around 3589.21 J/m^2^, which is 128.2% higher and 38.6% higher than that of DN-like structured sample 1# (poly(AAm-co-AA)/GEL_0.3_-Fe(III)) and sample 5# (poly(AAm-co-AA)/PVA_0.3_-Fe(III)), respectively. Through finding the superior feeding ratio of PVA and GEL, the shape recovery time of the poly(AAm-co-AA)/GEL/PVA-Fe(III) hydrogel could be further optimized to extremely approach that of sample 1#. When being stretched to λ_max_ = 5, sample 3# (poly(AAm-co-AA)/GEL_0.15_/PVA_0.15_-Fe(III)) with a “binary DN-like” structure could be fully recovered to its initial state within 25 min. It could also retain 100% of its initial toughness at λ_max_ of 5 after five successive loading–unloading tests (resting time of 5 min between two consecutive loading–unloading tests), while the corresponding data for sample 1# and sample 5# were 80% and 89%, respectively. Herein, the “binary DN-like” structure is a universal model for strong tough hydrogel design. Thus, we believed that many advanced strong tough hydrogels could be developed on basis of this theory in the future.

## Data Availability

The data presented in this study are available on request from the corresponding author.

## Supplementary Materials

The following are available online at https://www.mdpi.com/article/10.3390/polym15030724/s1. Figure S1: Schematic diagram showing the formation of the covalent/ionic crosslinked poly(AAm-co-AA)-Fe(III) network; Figure S2: The true water contents of the five samples; Figure S3: Representative loading-unloading profiles of the poly(AAm-co-AA)/GEL_0.3_-Fe(III) hydrogel (sample 1#) before and after UV radiation with λ_max_ of (A) 1.5 and (B) 3; Figure S4: Representative loading-unloading profiles of the poly(AAm-co-AA)/GEL_0.2_/PVA_0.1_-Fe(III) hydrogel (sample 2#) before and after UV radiation with λ_max_ of (A) 1.5 and (B) 3; Figure S5: Representative loading-unloading profiles of the poly(AAm-co-AA)/GEL_0.15_/PVA_0.15_-Fe(III) hydrogel (sample 3#) before and after UV radiation with λ_max_ of (A) 1.5 and (B) 3; Figure S6: Representative loading-unloading profiles of the poly(AAm-co-AA)/GEL_0.1_/PVA_0.2_-Fe(III) hydrogel (sample 4#) before and after UV radiation with λ_max_ of (A) 1.5 and (B) 3; Figure S7: Representative loading-unloading profiles of the poly(AAm-co-AA)/PVA_0.3_-Fe(III) hydrogel (sample 5#) before and after UV radiation with λ_max_ of (A) 1.5 and (B) 3; Figure S8: Cyclic loading–unloading tests of sample 1#–5#. Herein, for a certain group, the loading-unloading tests under different λ_max_ were carried out on different samples; Figure S9: Successive loading–unloading tests of sample 1#–5#. Herein, for a certain group, the loading-unloading tests under different λ_max_ were carried out on a same sample; Figure S10: Time-dependent toughness recovery capability of sample 1#; Figure S11: Time-dependent toughness recovery capability of sample 2#; Figure S12: Time-dependent toughness recovery capability of sample 3#; Figure S13: Time-dependent toughness recovery capability of sample 4#; Figure S14: Time-dependent toughness recovery capability of sample 5#; Figure S15: Comparisons about stress, Young’s modulus, and Uhys of sample 1# and sample 1# which was extended but given enough time for it to recover to its original shape; Figure S16: Comparisons about stress, Young’s modulus, and U_hys_ of sample 2# and sample 2# which was extended but given enough time for it to recover to its original shape; Figure S17: Comparisons about stress, Young’s modulus, and U_hys_ of sample 3# and sample 3# which was extended but given enough time for it to recover to its original shape; Figure S18: Comparisons about stress, Young’s modulus, and U_hys_ of sample 4# and sample 4# which was extended but given enough time for it to recover to its original shape; Figure S19: Comparisons about stress, Young’s modulus, and U_hys_ of sample 5# and sample 5# which was extended but given enough time for it to recover to its original shape; Figure S20: 5 consecutive loading-unloading tests of sample 1# with no resting time between two successive tests; Figure S21: 5 consecutive loading-unloading tests of sample 2# with no resting time between two successive tests; Figure S22: 5 consecutive loading-unloading tests of sample 3# with no resting time between two successive tests; Figure S23: 5 consecutive loading-unloading tests of sample 4# with no resting time between two successive tests; Figure S24: 5 consecutive loading-unloading tests of sample 5# with no resting time between two successive tests; Figure S25: 5 consecutive loading-unloading tests of sample 1# with 5 min’ resting time between two successive tests; Figure S26: 5 consecutive loading-unloading tests of sample 2# with 5 min’ resting time between two successive tests; Figure S27: 5 consecutive loading-unloading tests of sample 3# with 5 min’ resting time between two successive tests; Figure S28: 5 consecutive loading-unloading tests of sample 4# with 5 min’ resting time between two successive tests; Figure S29: 5 consecutive loading-unloading tests of sample 5# with 5 min’ resting time between two successive tests; Figure S30: 5 consecutive loading-unloading tests of sample 1# with 10 min’ resting time between two successive tests; Figure S31: 5 consecutive loading-unloading tests of sample 2# with 10 min’ resting time between two successive tests; Figure S32: 5 consecutive loading-unloading tests of sample 3# with 10 min’ resting time between two successive tests; Figure S33: 5 consecutive loading-unloading tests of sample 4# with 10 min’ resting time between two successive tests; Figure S34: 5 consecutive loading-unloading tests of sample 5# with 10 min’ resting time between two successive tests; Figure S35: 5 consecutive loading-unloading tests of sample 1# with 15 min’ resting time between two successive tests; Figure S36: 5 consecutive loading-unloading tests of sample 2# with 15 min’ resting time between two successive tests; Figure S37: 5 consecutive loading-unloading tests of sample 3# with 15 min’ resting time between two successive tests; Figure S38: 5 consecutive loading-unloading tests of sample 4# with 15 min’ resting time between two successive tests; Figure S39: 5 consecutive loading-unloading tests of sample 5# with 15 min’ resting time between two successive tests; Figure S40: 3D histogram summary showing the anti-fatigue capability of sample1#–5# (λ_max_ = 5, No resting time); Figure S41: 3D histogram summary showing the anti-fatigue capability of sample1#–5# (λ_max_ = 7, No resting time); Figure S42: 3D histogram summary showing the anti-fatigue capability of sample1#–5# (λ_max_ = 5, resting time of 5 min); Figure S43: 3D histogram summary showing the anti-fatigue capability of sample1#–5# (λ_max_ = 5, resting time of 5 min); Figure S44: 3D histogram summary showing the anti-fatigue capability of sample1#–5# (λ_max_ = 3, resting time of 10 min); Figure S45: 3D histogram summary showing the anti-fatigue capability of sample1#–5# (λ_max_ = 3, resting time of 15 min). Table S1: Fracture stress and U_hys_ at λ_max_ = 1.5 of sample 1#–5# before and after UV irradiation; Table S2: Fracture stress and U_hys_ at λ_max_ = 3 of sample 1#–5# before and after UV irradiation; Table S3: Young’s modulus, fracture energy, fracture stress, and fracture strain of sample 1#–5#; Table S4: A summary and comparison of energy dissipation capability of diverse energy dissipation models; Table S5: Tearing energy of sample 1#–5#; Table S6: A summary and comparison of toughness/shape capability of diverse energy dissipation models; Table S7: Shape recovery time of sample 1#–5#; Video S1: Shape recovery time of sample 1 with λ_max_ = 3; Video S2: Shape recovery time of sample 1 with λ_max_ = 5; Video S3: Shape recovery time of sample 1 with λ_max_ = 7; Video S4: Shape recovery time of sample 2 with λ_max_ = 3; Video S5: Shape recovery time of sample 2 with λ_max_ = 5; Video S6: Shape recovery time of sample 2 with λ_max_ = 7; Video S7: Shape recovery time of sample 3 with λ_max_ = 3; Video S8: Shape recovery time of sample 3 with λ_max_ = 5; Video S9: Shape recovery time of sample 3 with λ_max_ = 7; Video S10: Shape recovery time of sample 4 with λ_max_ = 3; Video S11: Shape recovery time of sample 4 with λ_max_ = 5; Video S12: Shape recovery time of sample 4 with λ_max_ = 7; Video S13: Shape recovery time of sample 5 with λ_max_ = 3; Video S14: Shape recovery time of sample 5 with λ_max_ = 5; Video S15: Shape recovery time of sample 5 with λ_max_ = 7. References [26,27,28,29,30,31,32,33,34,35,36] are cited in the Supplementary Materials.
