# Supplementary material for: Binary Double Network-like Structure: An Effective Energy-Dissipation System for Strong Tough Hydrogel Design
Source: Polymers (Basel). 2023 Jan 31;15(3):724. doi: 10.3390/polym15030724 (PMC9921367; doi:10.3390/polym15030724)
Supplement: Supplementary file 1 [file polymers-15-00724-s001.zip › polymers-2133881-supplementary.pdf]

Table S1 Specific feeding compositions of different groups

Sample	AAm (g)	AA (g)	PVA (g)	GEL (g)
**1# Poly(AAm-co-AA)/$GEL_{0.3}$-Fe(III)**	**1.71**	**0.091**	**0**	**0.3**
**2# Poly(AAm-co-AA)/$PVA_{0.1}$/$GEL_{0.2\text{-}Fe}$(III)**	**1.71**	**0.091**	**0.1**	**0.2**
**3# Poly(AAm-co-AA)/$PVA_{0.15}$/$GEL_{0.15}$-Fe(III)**	**1.71**	**0.091**	**0.15**	**0.15**
**4# Poly(AAm-co-AA)/$PVA_{0.2}$/$GEL_{0.1}$-Fe(III)**	**1.71**	**0.091**	**0.2**	**0.1**
**5# Poly(AAm-co-AA)/$PVA_{0.3}$-Fe(III)**	**1.71**	**0.091**	**0.3**	**0**

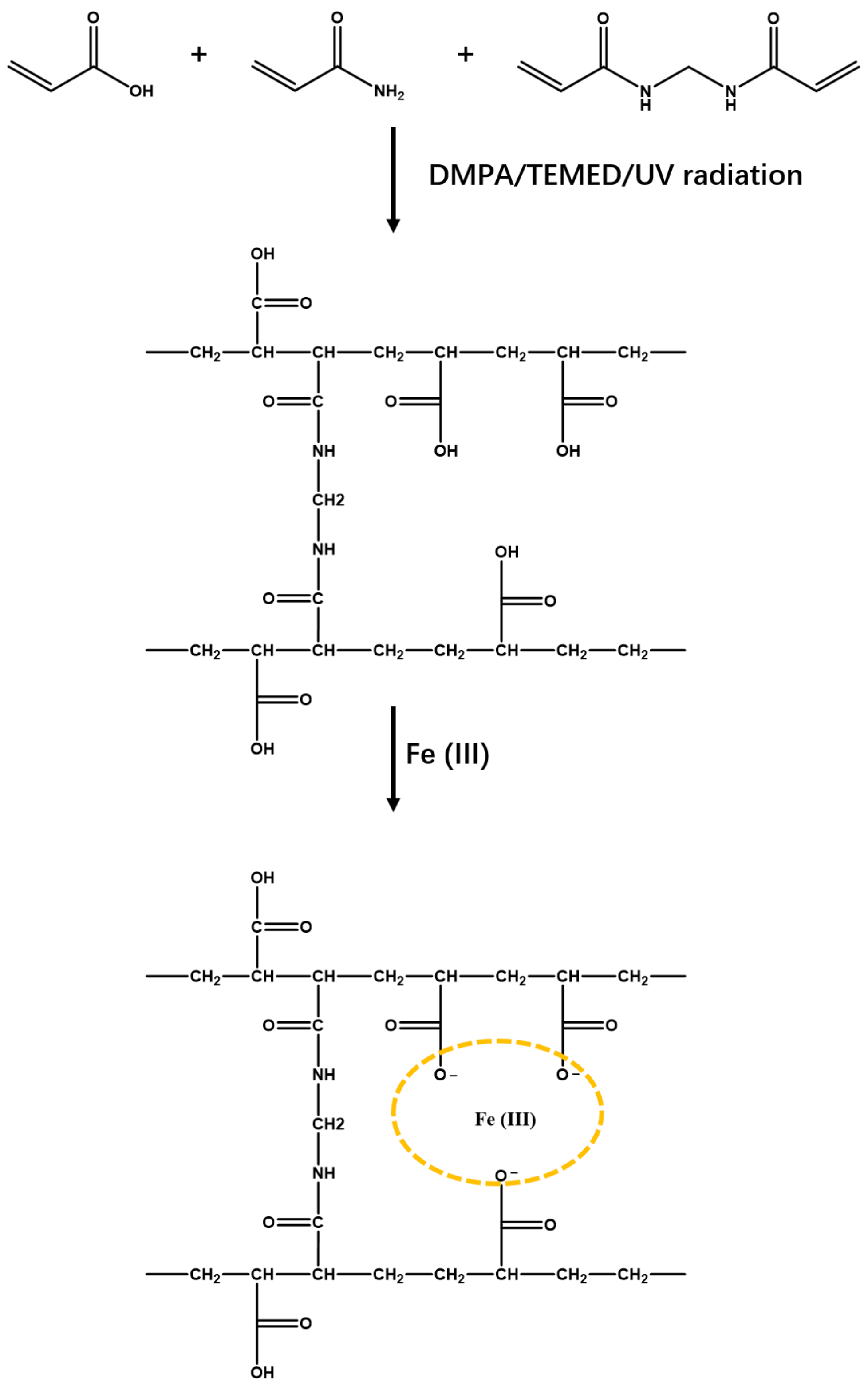

Figure S1 Schematic diagram showing the formation of the covalent/ionic crosslinked poly(AAm-co-AA)-Fe(III) network.

S1. Water Contents of 1# Poly(AAm-co-AA)/$GEL_{0.3}$-Fe(III), 2# Poly(AAm-co-AA)/$PVA_{0.1}$/$GEL_{0.2}$-Fe (III), 3# Poly(AAm-co-AA)/$PVA_{0.15}$/$GEL_{0.15}$-Fe(III), 4# Poly(AAm-co-AA)/$PVA_{0.2}$/$GEL_{0.1}$-Fe(III), and 5# Poly(AAm-co-AA)/$PVA_{0.3}$-Fe(III)

As-prepared sample was firstly weighed and recorded as m_0. Then the sample was placed in an oven (60 ℃) for 24 h to dehydrate it. Finally, the as-obtained dry sample was weighed again and recorded as m_1. The water content of the sample could be calculated using the following equation:

$$Water\ Content\ (\%) = \frac{m_0 - m_1}{m_1} \times 100\%$$

Five replicates were carried out for each group. Figure S2 demonstrates the water contents of 1#–5# samples

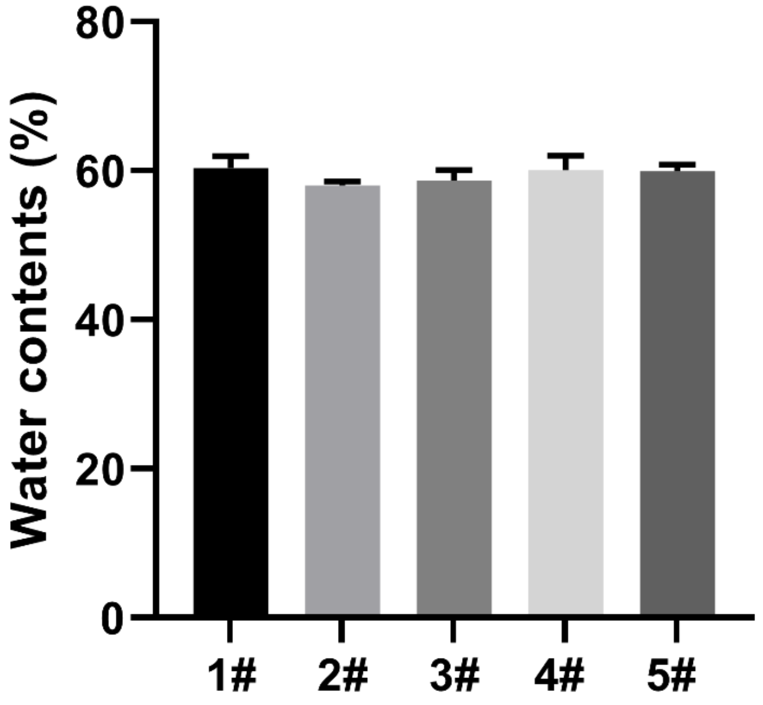

Figure S2 The water contents of 1#–5# samples.

S2 UV radiation induced reduction of Fe(III) to Fe(II) within the poly(AAm-co-AA)-Fe(III) network
Firstly, an as-prepared hydrogel sample was surface sprayed with 1 mL 0.2 mol/L citric acid solution, then the as-treated sample was subjected to UV radiation (254 nm) for 10 min until the color of the hydrogel was changed from brownish red to colorless. Then the hydrogel was quickly taken out and subjected to tensile test.

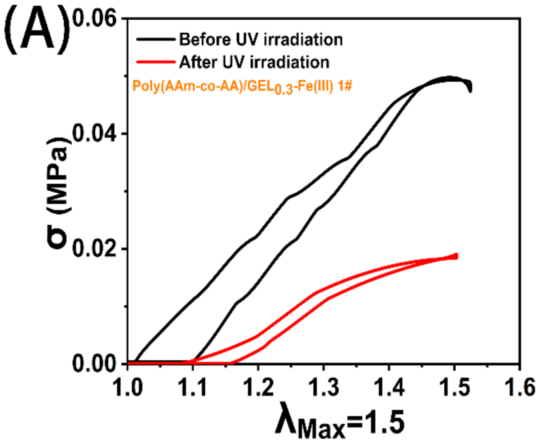

Figure S3 Representative loading-unloading profiles of the poly(AAm-co-AA)/$GEL_{0.3}$-Fe(III) hydrogel (sample 1#) before and after UV radiation with λ_{max} of (A) 1.5 and (B) 3.

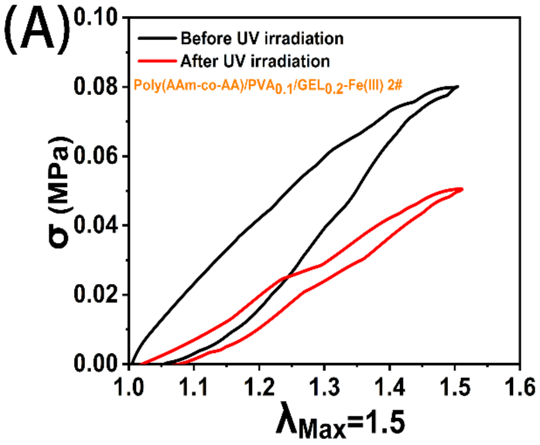

Figure S4 Representative loading-unloading profiles of the poly(AAm-co-AA)/$GEL_{0.2}$/$PVA_{0.1}$-Fe(III) hydrogel (sample 2#) before and after UV radiation with λ_{max} of (A) 1.5 and (B) 3.

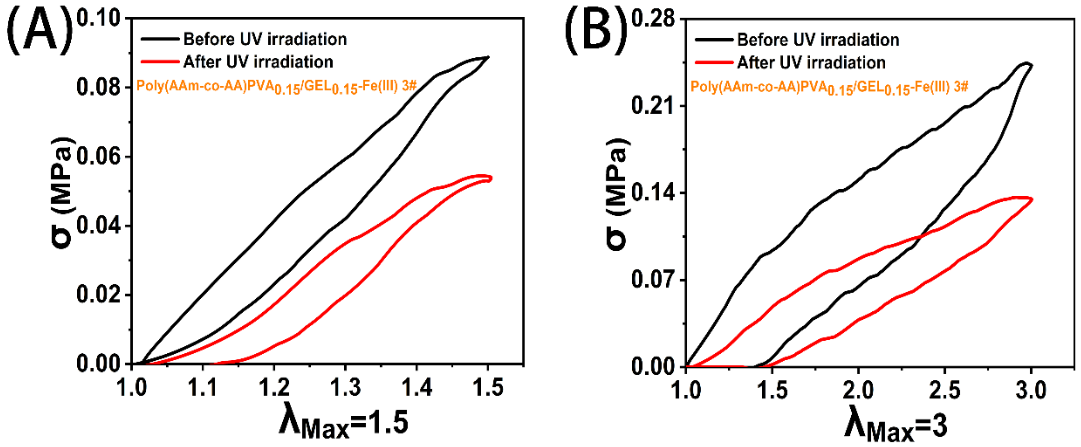

Figure S5 Representative loading-unloading profiles of the poly(AAm-co-AA)/$GEL_{0.15}$/$PVA_{0.15}$-Fe(III) hydrogel (sample 3#) before and after UV radiation with λ_{max} of (A) 1.5 and (B) 3.

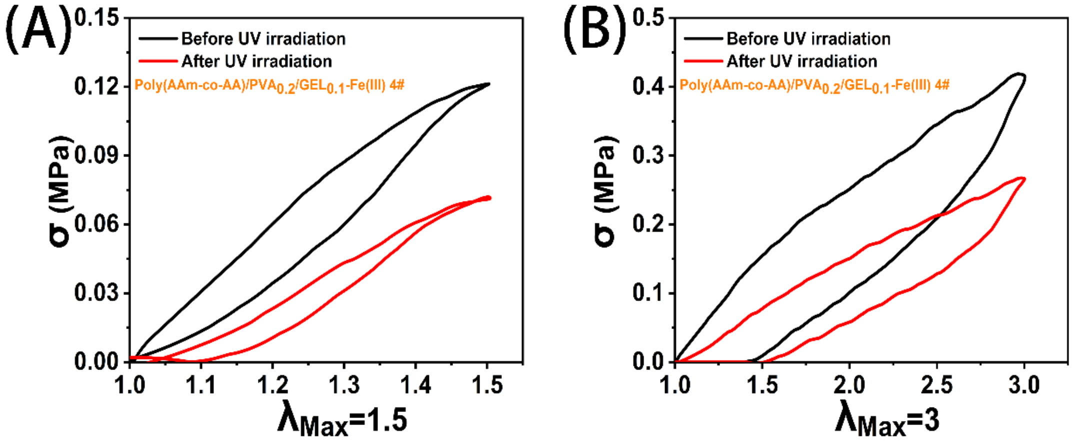

Figure S6 Representative loading-unloading profiles of the poly(AAm-co-AA)/$GEL_{0.1}$/$PVA_{0.2}$-Fe(III) hydrogel (sample 4#) before and after UV radiation with λ_{max} of (A) 1.5 and (B) 3.

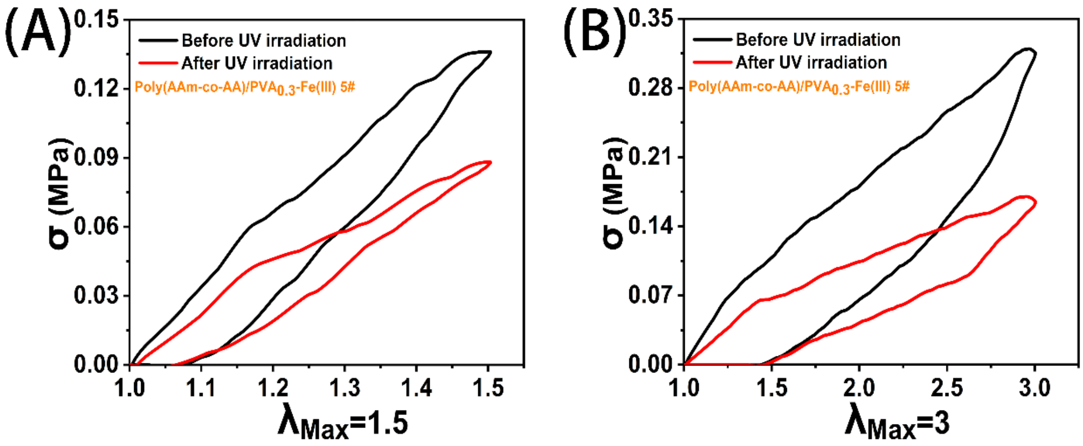

Figure S7 Representative loading-unloading profiles of the poly(AAm-co-AA)/$PVA_{0.3}$-Fe(III) hydrogel (sample 5#) before and after UV radiation with λ_{max} of (A) 1.5 and (B) 3.

Table S2 Fracture stress and U_{hys} at λ_{max}=1.5 of sample 1#–5# before and after UV irradiation

Sample (λ_{Max} = 1.5)	U_{hys} before UV irradiation （MJ/m^3）	U_{hys} after UV irradiation （MJ/m^3）	Stress before UV irradiation （MPa）	Stress after UV irradiation （MPa）
1# Poly(AAm-co-AA)/$GEL_{0.3}$-Fe(III)	0.025 ± 0.005	0.013 ± 0.005	0.049 ± 0.002	0.031 ± 0.011
2# Poly(AAm-co-AA)/$PVA_{0.1}$/$GEL_{0.2}$-Fe(III)	0.034 ± 0.006	0.027 ± 0.003	0.071 ± 0.007	0.056 ± 0.004
3# Poly(AAm-co-AA)/$PVA_{0.15}$/$GEL_{0.15}$-Fe(III)	0.041 ± 0.005	0.027 ± 0.006	0.094 ± 0.011	0.067 ± 0.006
4# Poly(AAm-co-AA)/$PVA_{0.2}$/$GEL_{0.1}$-Fe(III)	0.056 ± 0.009	0.036 ± 0.007	0.124 ± 0.014	0.079 ± 0.008
5# Poly(AAm-co-AA)/$PVA_{0.3}$-Fe(III)	0.059 ± 0.006	0.033 ± 0.010	0.129 ± 0.013	0.071 ± 0.014

Table S3 Fracture stress and U_{hys} at λ_{max}=3 of sample 1#–5# before and after UV irradiation

Sample (λ_{Max} = 3)	U_{hys} before UV irradiation (MJ/m^3)	U_{hys} after UV irradiation (MJ/m^3)	Stress before UV irradiation (MPa)	Stress after UV irradiation (MPa)
1# Poly(AAm-co-AA)/$GEL_{0.3}$-Fe(III)	0.029 ± 0.010	0.223 ± 0.040	0.161 ± 0.003	0.126 ± 0.017
2# Poly(AAm-co-AA)/$PVA_{0.1}$/$GEL_{0.2}$-Fe(III)	0.353 ± 0.021	0.267 ± 0.006	0.196 ± 0.013	0.158 ± 0.005
3# Poly(AAm-co-AA)/$PVA_{0.15}$/$GEL_{0.15}$-Fe(III)	0.387 ± 0.061	0.277 ± 0.035	0.214 ± 0.031	0.156 ± 0.016
4# Poly(AAm-co-AA)/$PVA_{0.2}$/$GEL_{0.1}$-Fe(III)	0.903 ± 0.391	0.527 ± 0.078	0.547 ± 0.225	0.332 ± 0.047
5# Poly(AAm-co-AA)/$PVA_{0.3}$-Fe(III)	0.504 ± 0.037	0.387 ± 0.081	0.276 ± 0.013	0.234 ± 0.027

Table S4 Shape recovery time of sample 1#–5#.

Sample	Recovery time (λ_{Max}=3, min)	Recovery time (λ_{Max}=5, min)	Recovery time (λ_{Max}=7, min)
1# Poly(AAm-co-AA)/$GEL_{0.3}$-Fe(III)	9 ± 0.58	15 ± 1.15	24 ± 2.52
2# Poly(AAm-co-AA)/$PVA_{0.1}$/$GEL_{0.2}$-Fe(III)	10 ± 1.00	24 ± 1.53	36 ± 4.00
3# Poly(AAm-co-AA)/$PVA_{0.15}$/$GEL_{0.15}$-Fe(III)	11 ± 1.53	27 ± 2.52	37 ± 0.58
4# Poly(AAm-co-AA)/$PVA_{0.2}$/$GEL_{0.1}$-Fe(III)	17 ± 1.15	41 ± 3.61	52 ± 2.65
5# Poly(AAm-co-AA)/$PVA_{0.3}$-Fe(III)	32 ± 4.73	47 ± 4.36	66 ± 4.16

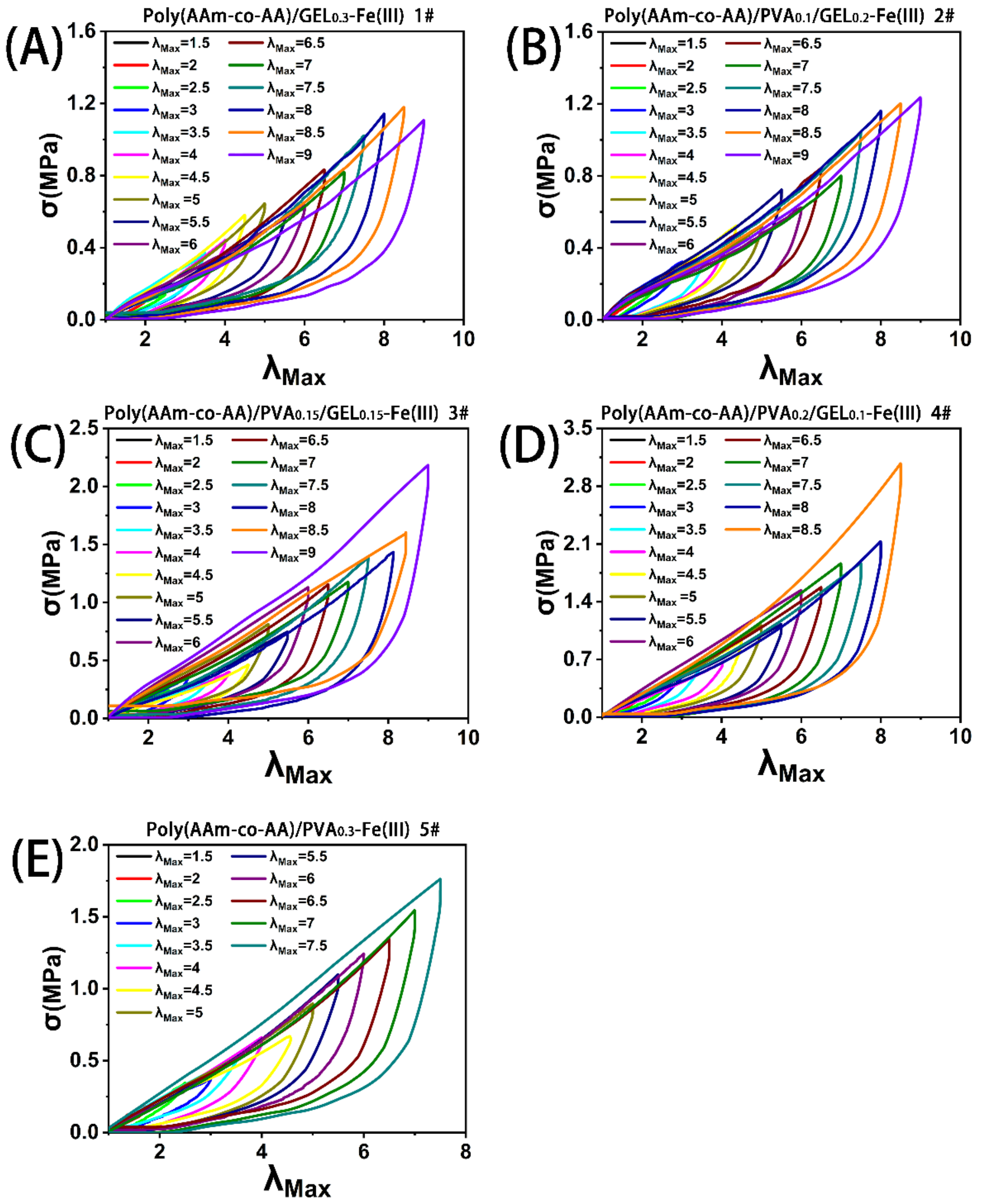

Figure S8 Cyclic loading–unloading tests of sample 1#–5#. (A), (B), (C), (D), and (E) are corresponded to sample 1#, 2#, 3#, 4#, and 5#, respectively. Herein, for a certain group, the loading-unloading tests under different λ_{max} were carried out on different samples.

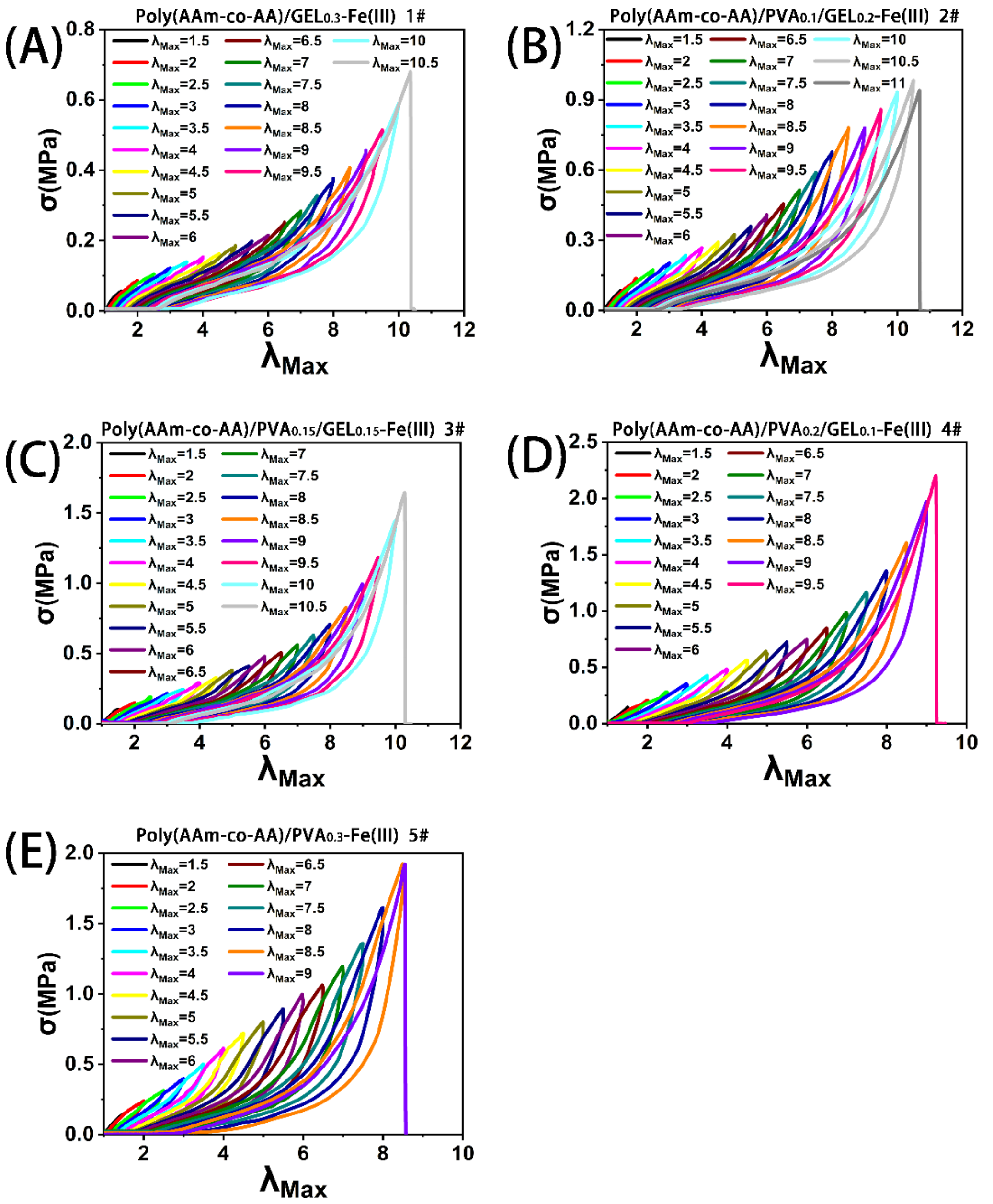

Figure S9 Successive loading–unloading tests of sample 1#–5#. (A), (B), (C), (D), and (E) are corresponded to sample 1#, 2#, 3#, 4#, and 5#, respectively. Herein, for a certain group, the loading-unloading tests under different λ_{max} were carried out on a same sample.

Table S5 Young's modulus, fracture energy, fracture stress, and fracture strain of sample 1#–5#.

Sample	Average Young's modulus (MPa)	Average fracture energy ($J \cdot m^{-2}$)	Average fracture stress (Mpa)	Average fracture strain （mm）
1# Poly(AAm-co-AA)/$GEL_{0.3}$-Fe(III)	0.04 ± 0.01	91985 ± 1539	0.96 ± 0.02	9.69 ± 0.28
2# Poly(AAm-co-AA)/$PVA_{0.1}$/$GEL_{0.2\text{-}Fe}$(III)	0.06±0.01	160955 ± 58042	1.25 ± 0.09	9.88 ± 0.38
3# Poly(AAm-co-AA)/$PVA_{0.15}$/$GEL_{0.15}$-Fe(III)	0.07±0.02	175840 ± 14584	1.63 ± 0.03	9.60 ± 0.16
4# Poly(AAm-co-AA)/$PVA_{0.2}$/$GEL_{0.1}$-Fe(III)	0.11 ± 0.02	219063 ± 49269	2.08 ± 0.36	9.15 ± 0.18
5# Poly(AAm-co-AA)/$PVA_{0.3}$-Fe(III)	0.19 ± 0.01	161971 ± 7394.50	1.89 ± 0.01	6.97 ± 0.59

Table S6 A summary and comparison of energy dissipation capability of diverse energy dissipation models

Dissipated energy			
Type	λ_{max}	U_{hys}(J/m^3)	References
Ionically-crosslinked dual-network hydrogels (PNAGA/CMC-Fe)	3	12.3×10^6	*Polymer* [1]
Ionically-crosslinked dual-network hydrogels (Agar/PAMAAc-Fe^{3+} DN gel)	10	2.3×10^6	*Chemistry of Materials* [2]
Double network hydrogel (PAM-CS-S DN hydrogels)	3	1.3×10^6	*Advanced Materials* [3]
Micellar-incorporated hydrogels poly(AM-co-MA/SP)	4	3.6×10^5	*Journal of Materials Chemistry C* [4]
Ionically linked hydrogels (Poly(PAAm-co-AA)-Fe^{3+})	5	1.2×10^7	*Advanced Materials* [5]
Hydrophobic interaction hydrogel (HM35P10)	6	11.7×10^6	*Soft Matter [6]*
Micellar-incorporated hydrogels (poly-HEAA 3 -MA 10 @T80 0.73)	7	1.4×10^5	*Jouranl of Polymer Science Part B-Polymer Physics [7]*
Multiple hydrogen bonds (PAPA0.04 -PAM3)	10	3.4×10^4	*Biomacromolecules [8]*
Multiple hydrogen bonds (PVA-TA300)	3	1.1×10^6	*Macromolecules [9]*

Double network hydrogel (Gelatin/PAAm gels）	30	1.0×10^6	*Journal of Materials Chemistry B [10]*
Double network hydrogel （Gelatin/PAAm gel）	8	16.6×10^3	*Soft Matter [11]*
Triple network hydrogel (Gelatin/k-carrageenan/PAAm gel)	8	33.7×10^4	*Soft Matter [11]*

Table S7 Tearing energy of sample 1#–5#.

Sample	F_{avf} (N)	$2F_{avf}$ /B (J/m^2)
1# Poly(AAm-co-AA)/$GEL_{0.3}$-Fe(III)	1.53±0.24	1537±66
2# Poly(AAm-co-AA)/$PVA_{0.1}$/$GEL_{0.2\text{-}Fe}$(III)	1.98±0.05	1962±18
3# Poly(AAm-co-AA)/$PVA_{0.15}$/$GEL_{0.15}$-Fe(III)	2.74±0.48	2869±233
4# Poly(AAm-co-AA)/$PVA_{0.2}$/$GEL_{0.1}$-Fe(III)	3.56±0.48	3589±580
5# Poly(AAm-co-AA)/$PVA_{0.3}$-Fe(III)	2.52±0.22	2589±113

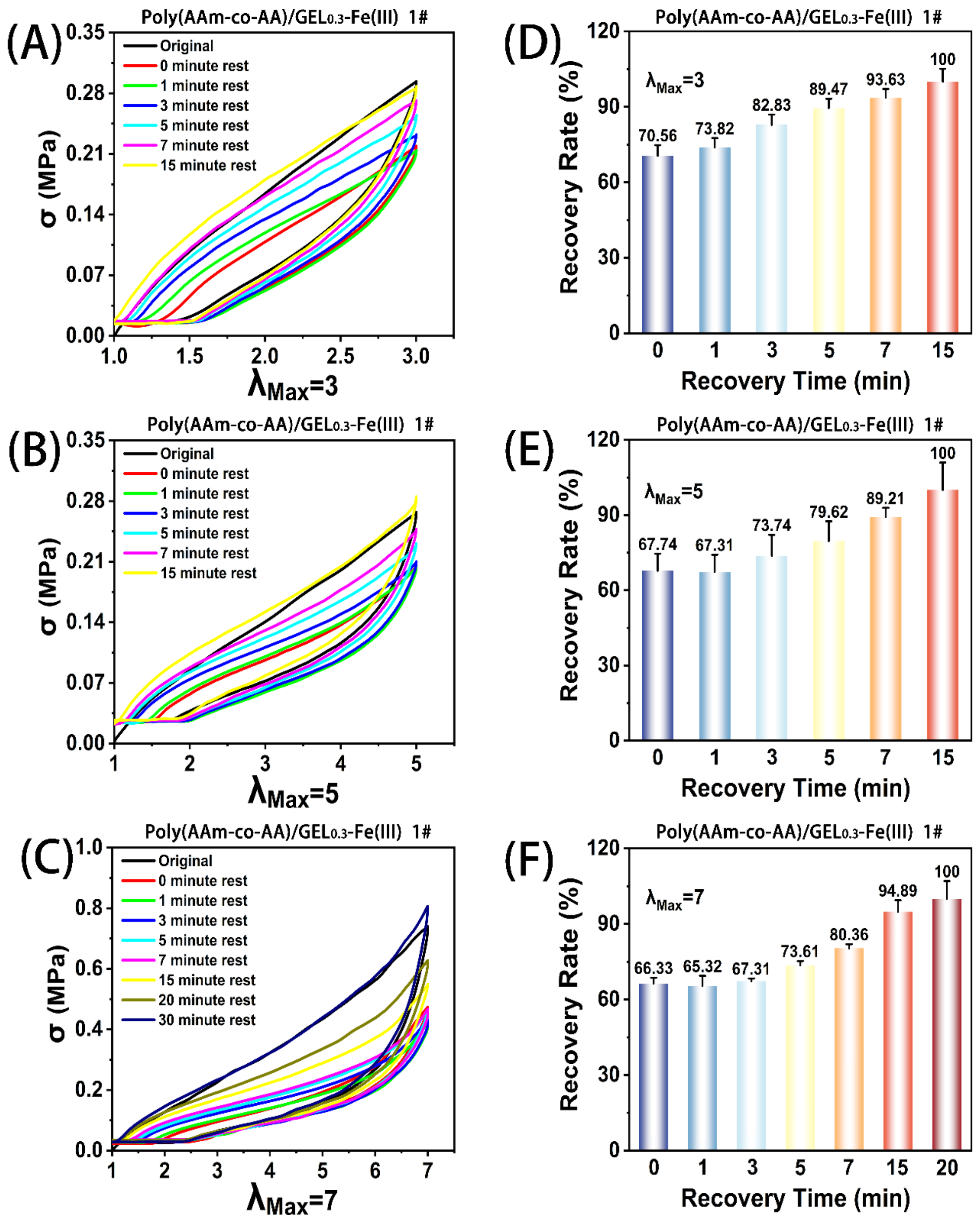

Figure S10 Time-dependent toughness recovery capability of sample 1#. Pristine stress-strain curves of (A) λ_{max} = 3, (B) λ_{max} = 5, and (C) λ_{max} = 7. Histograms of toughness recovery rate of (D) λ_{max} = 3, (E) λ_{max} = 5, (F) λ_{max} = 7.

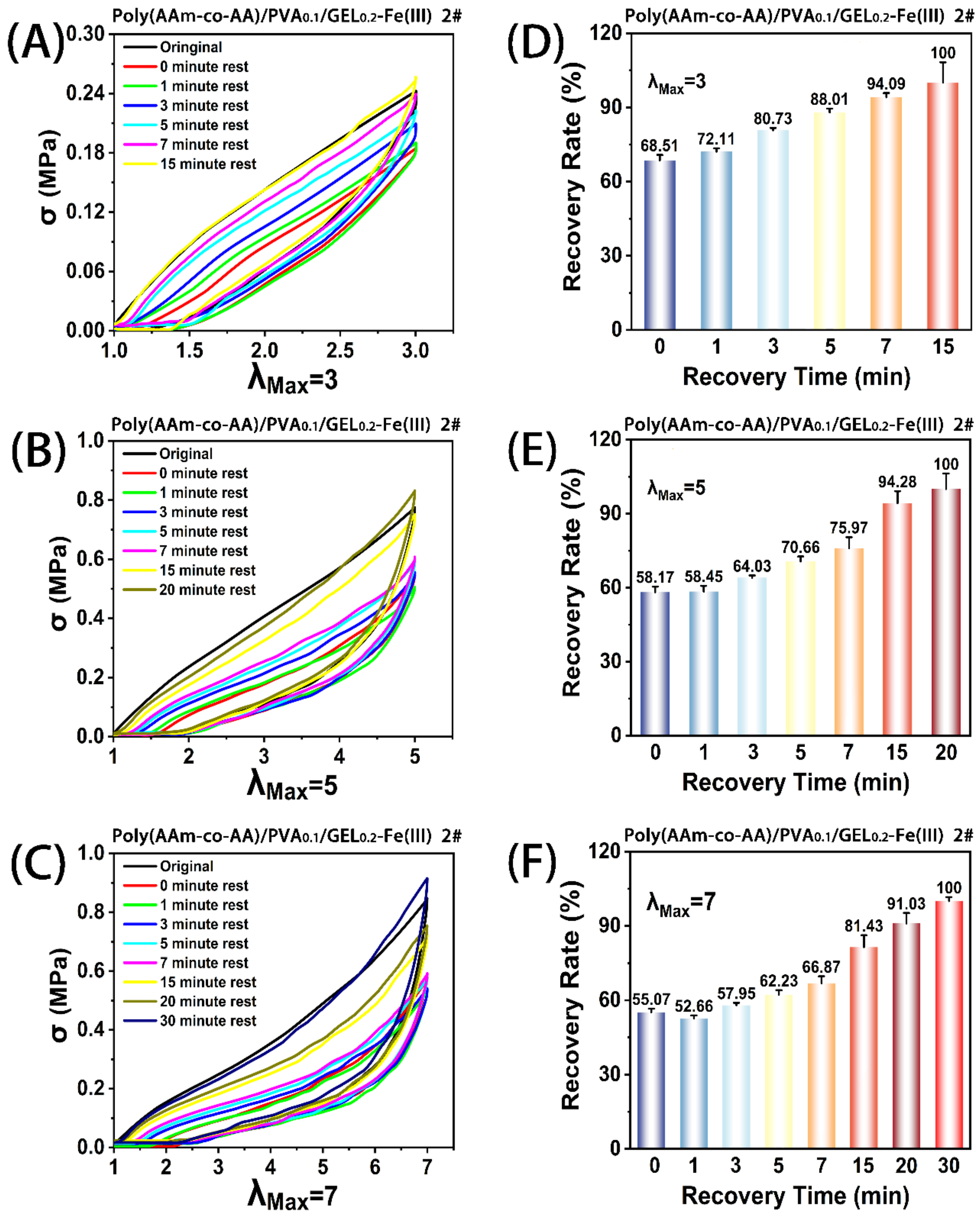

Figure S11 Time-dependent toughness recovery capability of sample 2#. Pristine stress-strain curves of (A) λ_{max} = 3, (B) λ_{max} = 5, and (C) λ_{max} = 7. Histograms of toughness recovery rate of (D) λ_{max} = 3, (E) λ_{max} = 5, (F) λ_{max} = 7.

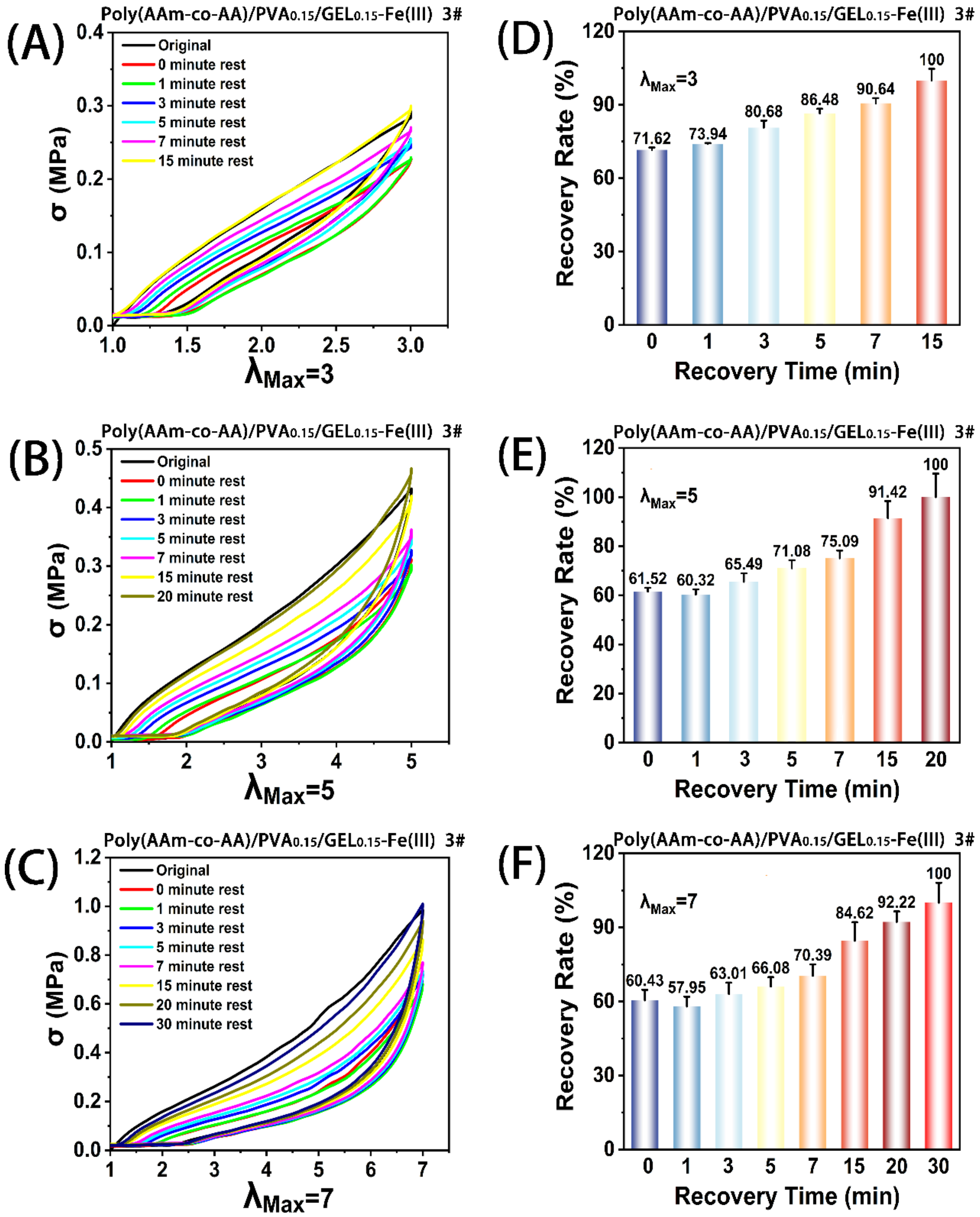

Figure S12 Time-dependent toughness recovery capability of sample 3#. Pristine stress-strain curves of (A) λ_{max} = 3, (B) λ_{max} = 5, and (C) λ_{max} = 7. Histograms of toughness recovery rate of (D) λ_{max} = 3, (E) λ_{max} = 5, (F) λ_{max} = 7.

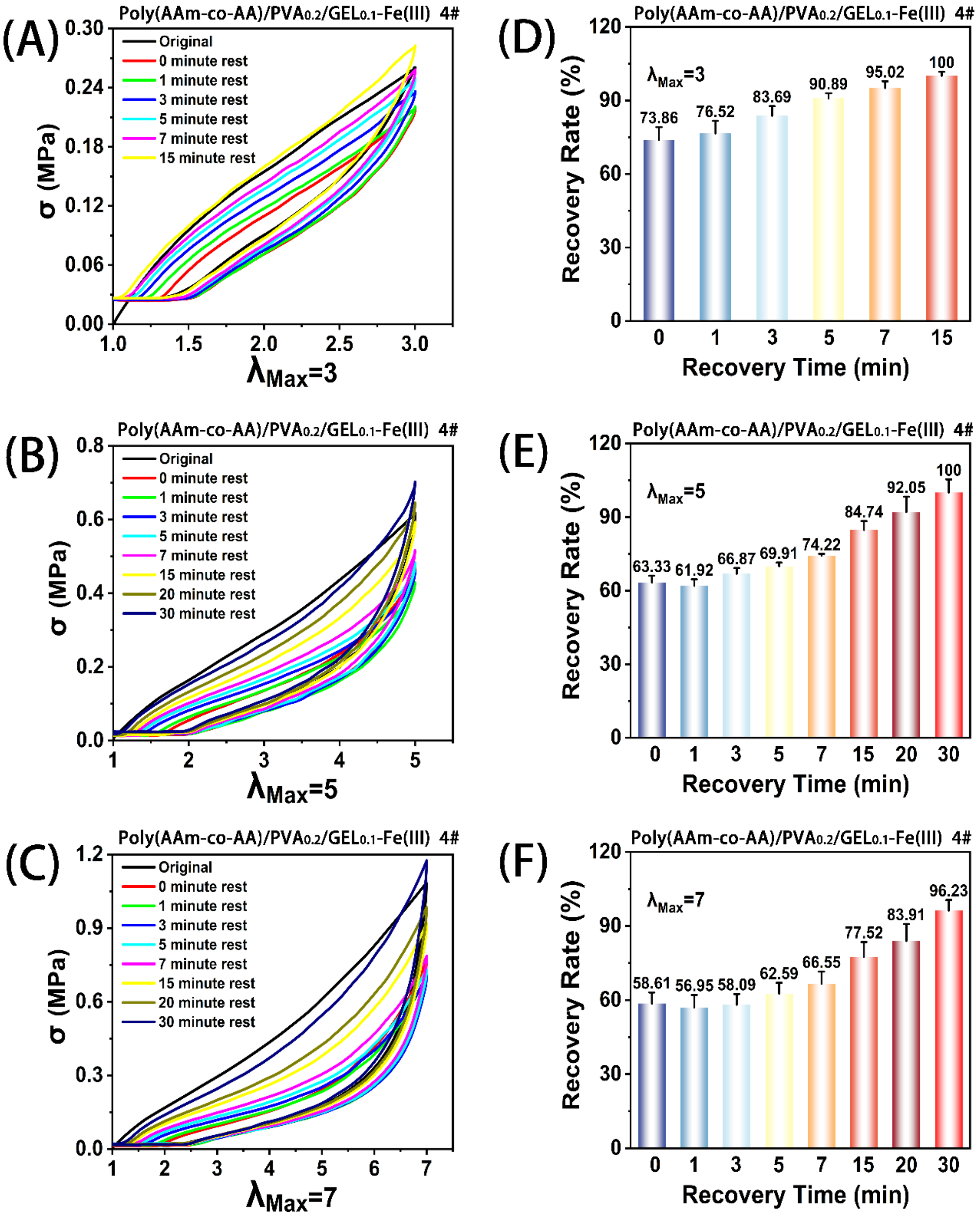

Figure S13 Time-dependent toughness recovery capability of sample 4#. Pristine stress-strain curves of (A) λ_{max} = 3, (B) λ_{max} = 5, and (C) λ_{max} = 7. Histograms of toughness recovery rate of (D) λ_{max} = 3, (E) λ_{max} = 5, (F) λ_{max} = 7.

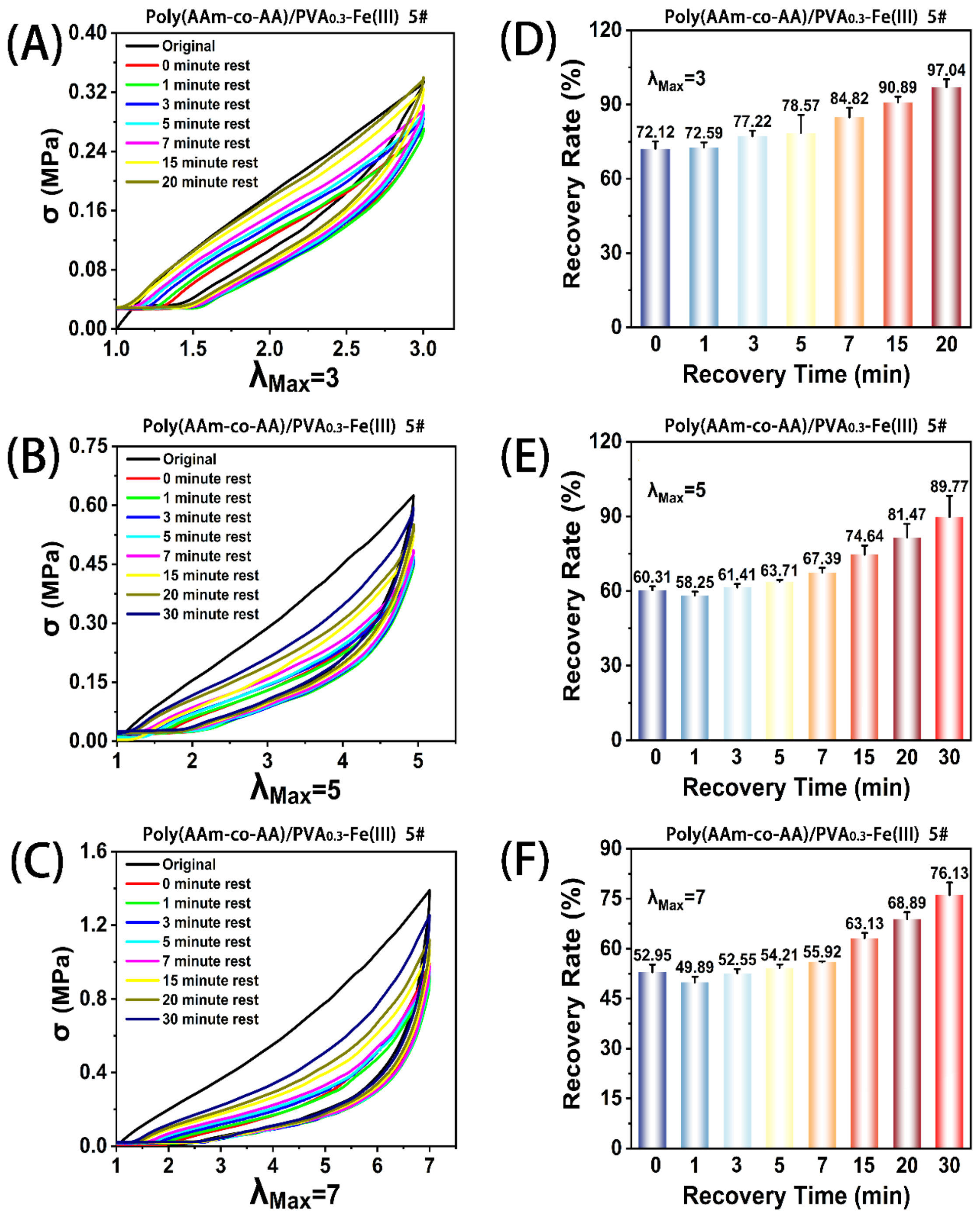

Figure S14 Time-dependent toughness recovery capability of sample 5#. Pristine stress-strain curves of (A) λ_{max} = 3, (B) λ_{max} = 5, and (C) λ_{max} = 7. Histograms of toughness recovery rate of (D) λ_{max} = 3, (E) λ_{max} = 5, (F) λ_{max} = 7.

Table S8 A summary and comparison of toughness/shape capability of diverse energy dissipation models

Type	λ_{max}	Recovery time (min)	References
Block copolymer hydrogel (B-DN gel)	2	5 min (Only restores 85 % of the toughness of the material)	*Advanced Materials* [12]
Ionically linked hydrogels (Alg/PAAm-Ca^{2+})	7	After storing at 80 °C for 1 day, the work on reloading was recovered to 74 % of that of the first loading.	*Nature* [13]
Micellar-incorporated hydrogels (Hydrogels with F127DA micelles)	11	This 120 % residual strain could spontaneously but partly recover at 25 °C for about 30 h, leaving an unrecoverable residual strain of about 20 %	*ACS Macro Letters* [14]
Ionically-crosslinked dual-network hydrogels (Agar/PAMAAc-Fe^{3+} DN gel)	6	After 20 min of resting, the gels can recover~95 % of toughness.	*Chemistry of Materials* [2]
Ionically linked hydrogels (Poly(PAAm-co-AA)-Fe^{3+})	4	When the recovery time was 4 h, elastic modulus recovered to its original state, and the hysteresis loop area recovered to 87.6 % of its original value.	*Advanced Materials* [5]
Hydrophobic interaction hydrogel (HM35P10)	2.5	≈4 h at room temperature to 70.8 %, at 2.15 MPa stress, and 150% strain	*Soft Matter [6]*
Multiple hydrogen bonds ($PAPA_{0.04}$-PAM_3)	6	The recovery ratio of dissipated energy gradually increased and could reach 85 % after resting for 20 min, demonstrating the recombination of hydrogen bonds and promising self-recovery behavior.	*Biomacromolecules* [8]

Multiple hydrogen bonds (PVA−TA300(S) gel)	2	They recovered to nearly 68 % after 12 h when the strain was 100 %	*Macromolecules* [9]
Double network hydrogel (Gelatin/PAAm gels）	10	After 10 min. heating treatment at different temperatures, hysteresis loops became larger and energy dissipation increased as well. Quantitatively, toughness recovery rate, defined by the ratio of U_{hys} at different temperatures to U_{hys} at the first loading, was 71 %, 87 %, 88 %, 90 %, and 95 % at heating temperature of 15, 30, 40, 50, and 60 ℃, respectively	*Journal of Materials Chemistry B* [10]

Double network hydrogel (PU/DHIR-0-20; PU/DHIR0.150-20; PU/DHIR-0.244-20 hydrogel)	4	The hysteresis loops of the PU/DHIR-0-20 hydrogel show complete recovery to the original state after 10 min at room temperature. As the cross-linker content in second network increases, the time required for complete recovery becomes longer. Specifically, the PU/DHIR0.150-20 hydrogel requires 40 min to completely recover, and the PU/DHIR-0.244-20 hydrogel requires almost 6 h for full recovery.	*ACS Applied Materials & Interfaces* [15]
Ionically-crosslinked dual-network hydrogels (PNAGA/CMC-Fe)	3	After standing for 12 h, the dissipated energy and Young’s modulus of the stretched DN gel sample approach to those of the original one, and the recovery ratios of toughness (dissipated energy) and stiffness (Young’s modulus) achieve as high as 84.9 % and 87.7 %, respectively, signifying the reconstruction of reversible physical crosslinks before reloading.	*Polymer* [1]

Double network hydrogel (PAM-CS-A DN hydrogels)	3	We applied the ratio of work (W 2nd/W 1st) performed in two tensile tests as recovery efficiency, and discovered that the recovery efficiencies of PAM-CS-A could exceed more than 95 %, respectively, after relaxing for 4 h	*Advanced Materials* [3]
Triple network hydrogel (gelatin/k-carrageenan/PAAm gels）	8	A 61.03 % recovery of energy dissipation and 86.96 % recovery of elastic modulus are obtained when storing the deformed sample at 60 ℃ for 30 min.	*Soft Matter* [11]

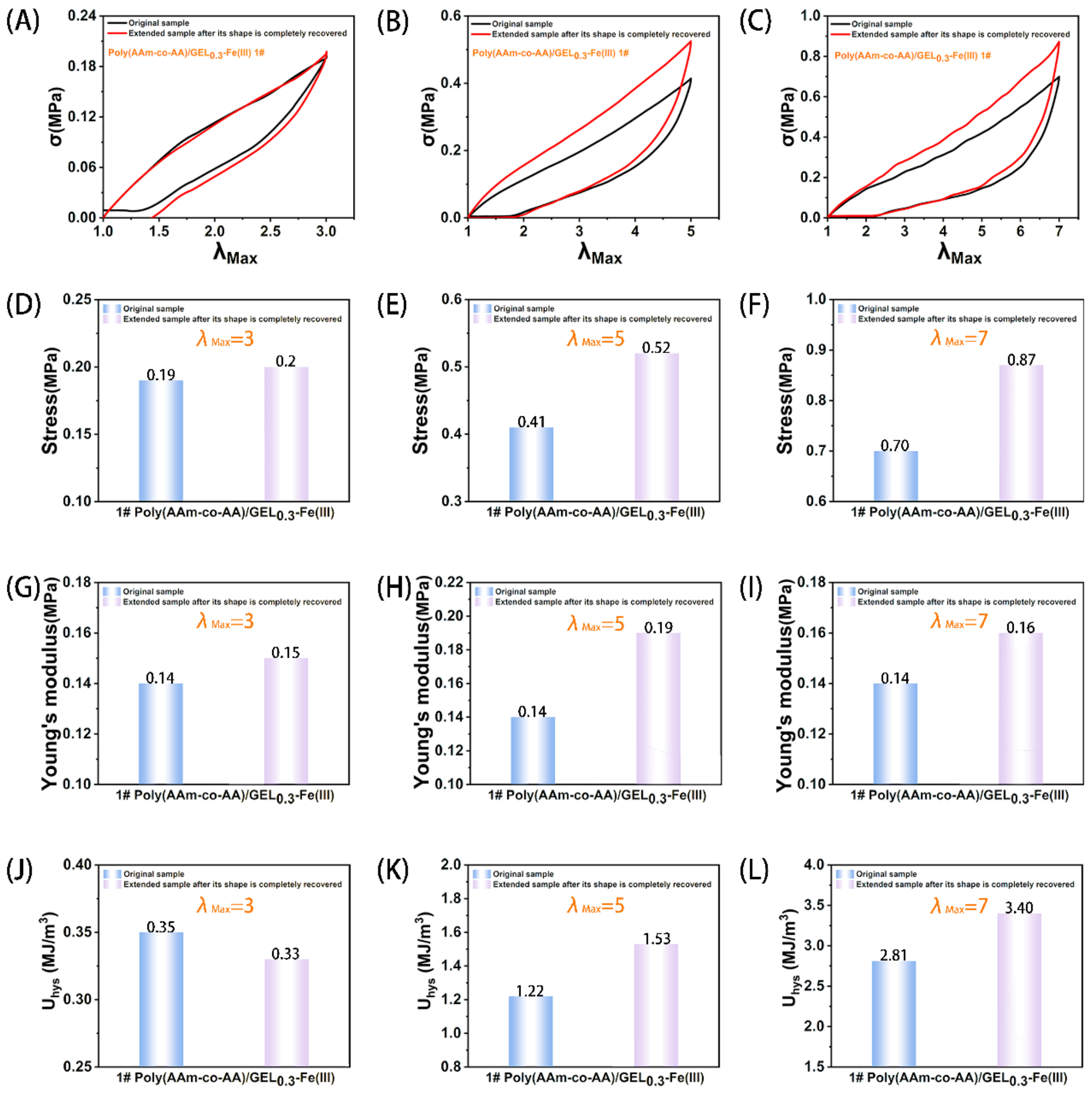

Figure S15 Comparisons about stress, Young's modulus, and U_{hys} of sample 1# and sample 1# which was extended but given enough time for it to recover to its original shape. Pristine stress-strain curves of (A) λ_{max} = 3, (B) λ_{max} = 5, and (C) λ_{max} = 7. Histograms of fracture stress, Young's modulus, and U_{hys} of (D, G, and J) λ_{max} = 3, (E, H, and K) λ_{max} = 5, (F, I, and L) λ_{max} = 7.

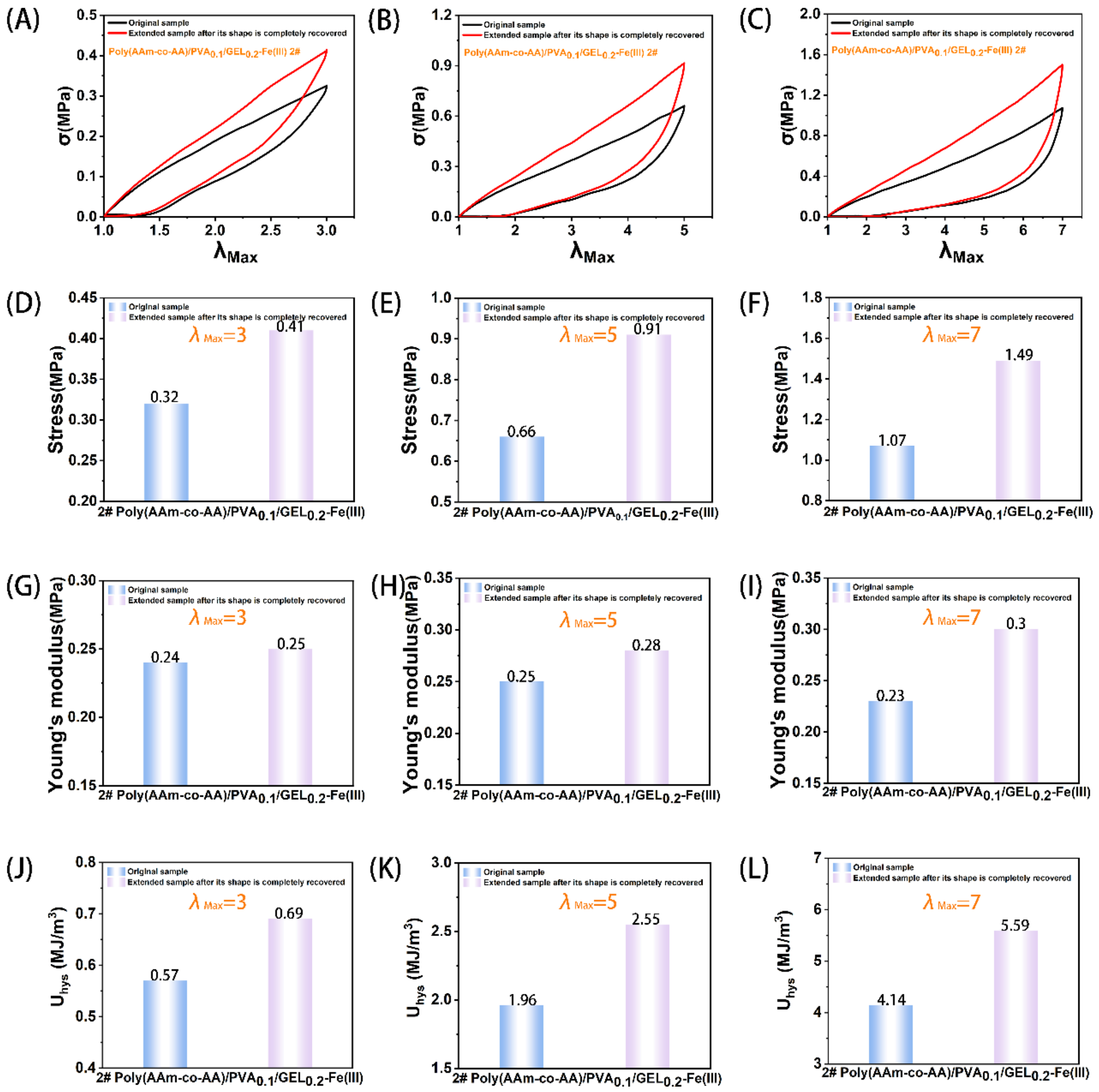

Figure S16 Comparisons about stress, Young's modulus, and U_{hys} of sample 2# and sample 2# which was extended but given enough time for it to recover to its original shape. Pristine stress-strain curves of (A) λ_{max} = 3, (B) λ_{max} = 5, and (C) λ_{max} = 7. Histograms of fracture stress, Young's modulus, and U_{hys} of (D, G, and J) λ_{max} = 3, (E, H, and K) λ_{max} = 5, (F, I, and L) λ_{max} = 7.

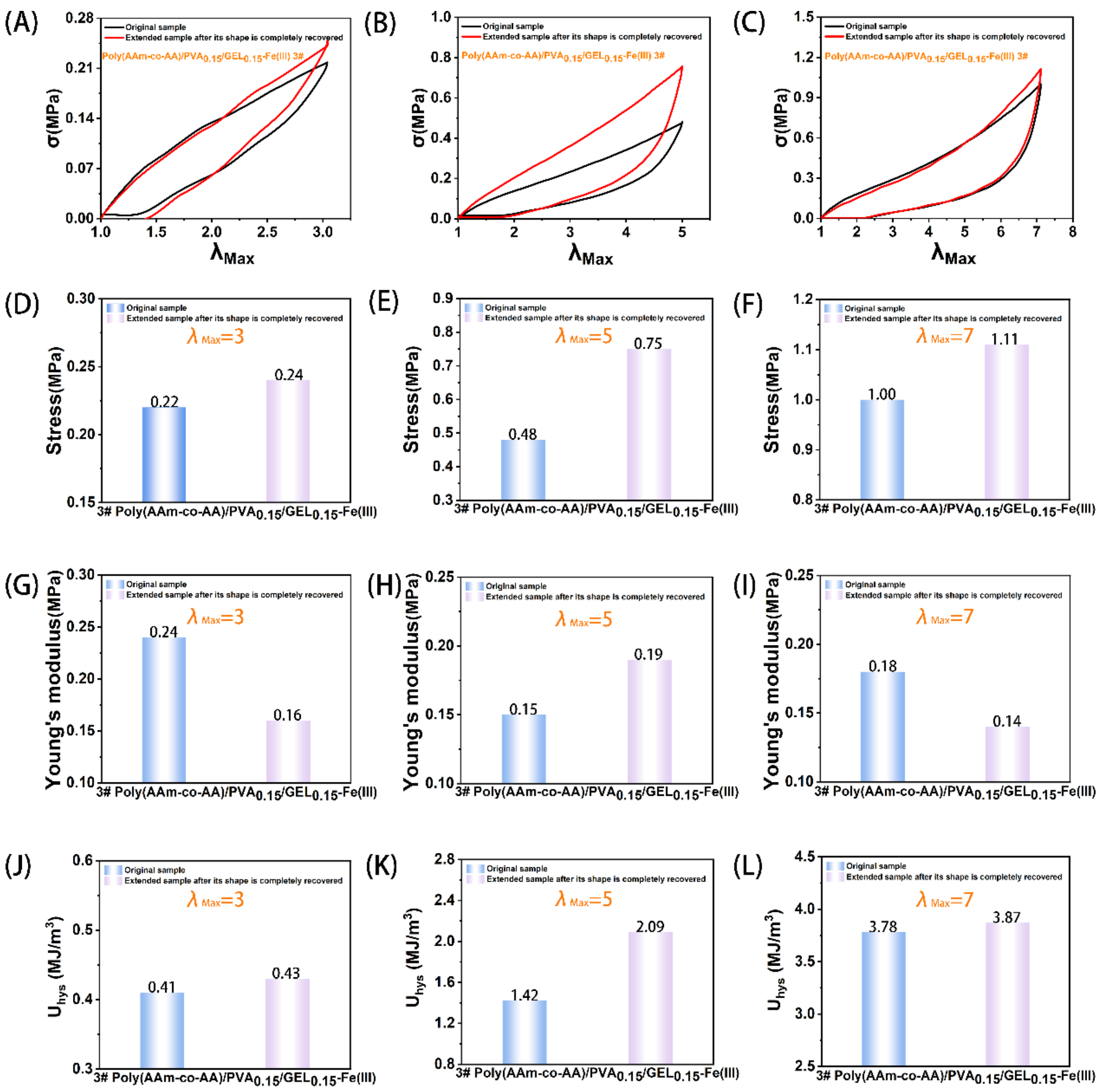

Figure S17 Comparisons about stress, Young's modulus, and U_{hys} of sample 3# and sample 3# which was extended but given enough time for it to recover to its original shape. Pristine stress-strain curves of (A) λ_{max} = 3, (B) λ_{max} = 5, and (C) λ_{max} = 7. Histograms of fracture stress, Young's modulus, and U_{hys} of (D, G, and J) λ_{max} = 3, (E, H, and K) λ_{max} = 5, (F, I, and L) λ_{max} = 7.

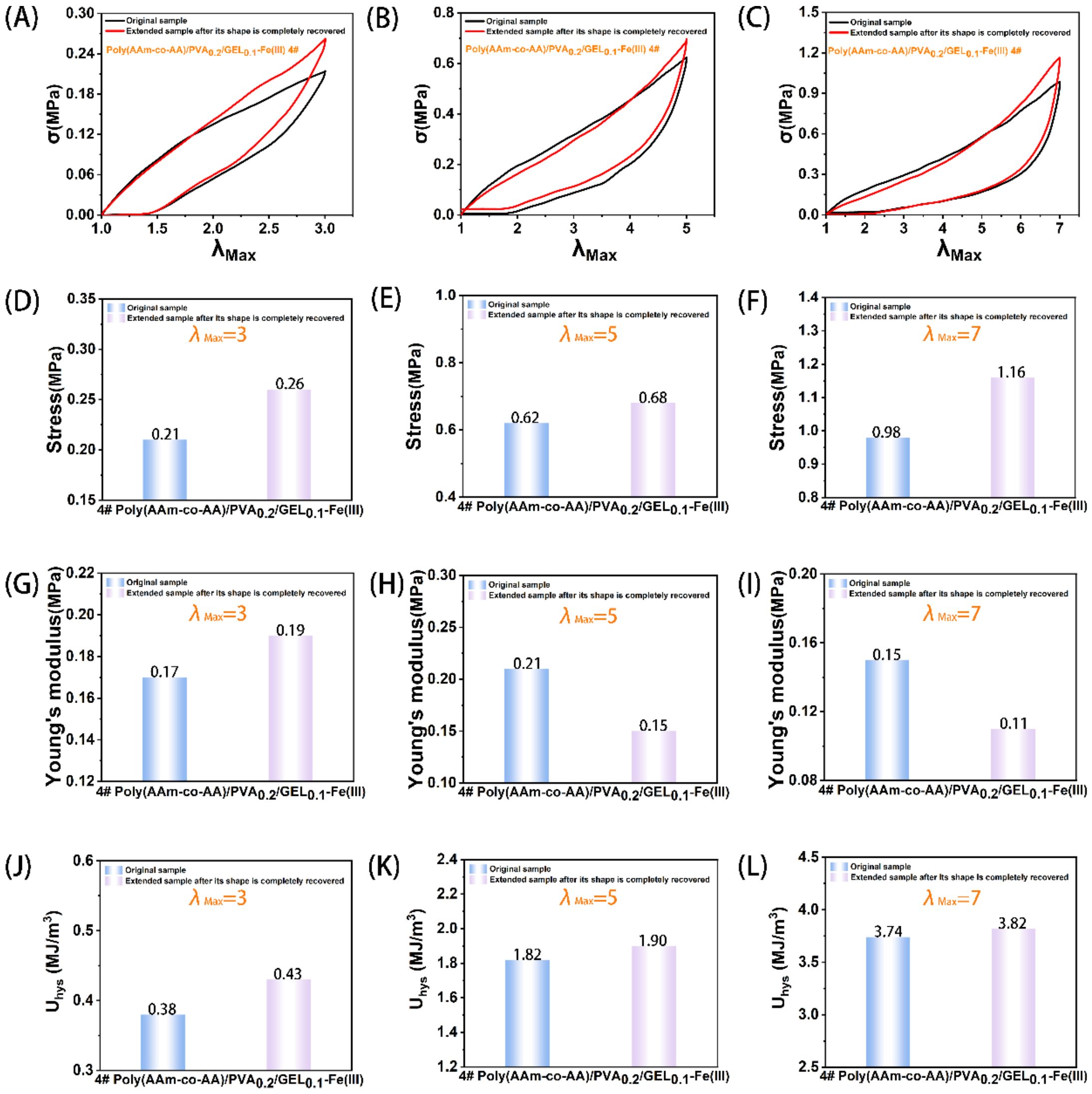

Figure S18 Comparisons about stress, Young's modulus, and U_{hys} of sample 4# and sample 4# which was extended but given enough time for it to recover to its original shape. Pristine stress-strain curves of (A) λ_{max} = 3, (B) λ_{max} = 5, and (C) λ_{max} = 7. Histograms of fracture stress, Young's modulus, and U_{hys} of (D, G, and J) λ_{max} = 3, (E, H, and K) λ_{max} = 5, (F, I, and L) λ_{max} = 7.

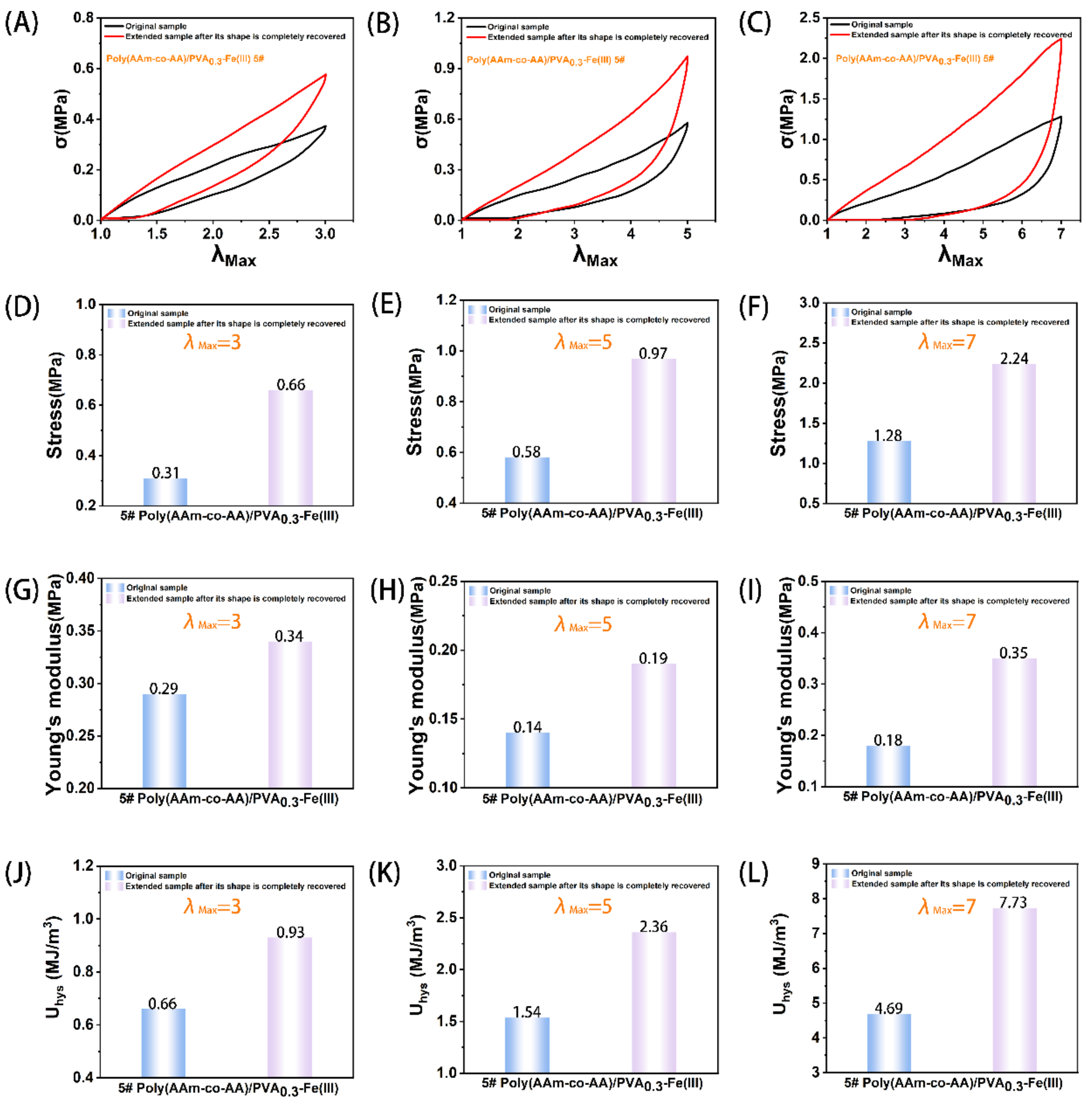

Figure S19 Comparisons about stress, Young's modulus, and U_{hys} of sample 5# and sample 5# which was extended but given enough time for it to recover to its original shape. Pristine stress-strain curves of (A) λ_{max} = 3, (B) λ_{max} = 5, and (C) λ_{max} = 7. Histograms of fracture stress, Young's modulus, and U_{hys} of (D, G, and J) λ_{max} = 3, (E, H, and K) λ_{max} = 5, (F, I, and L) λ_{max} = 7.

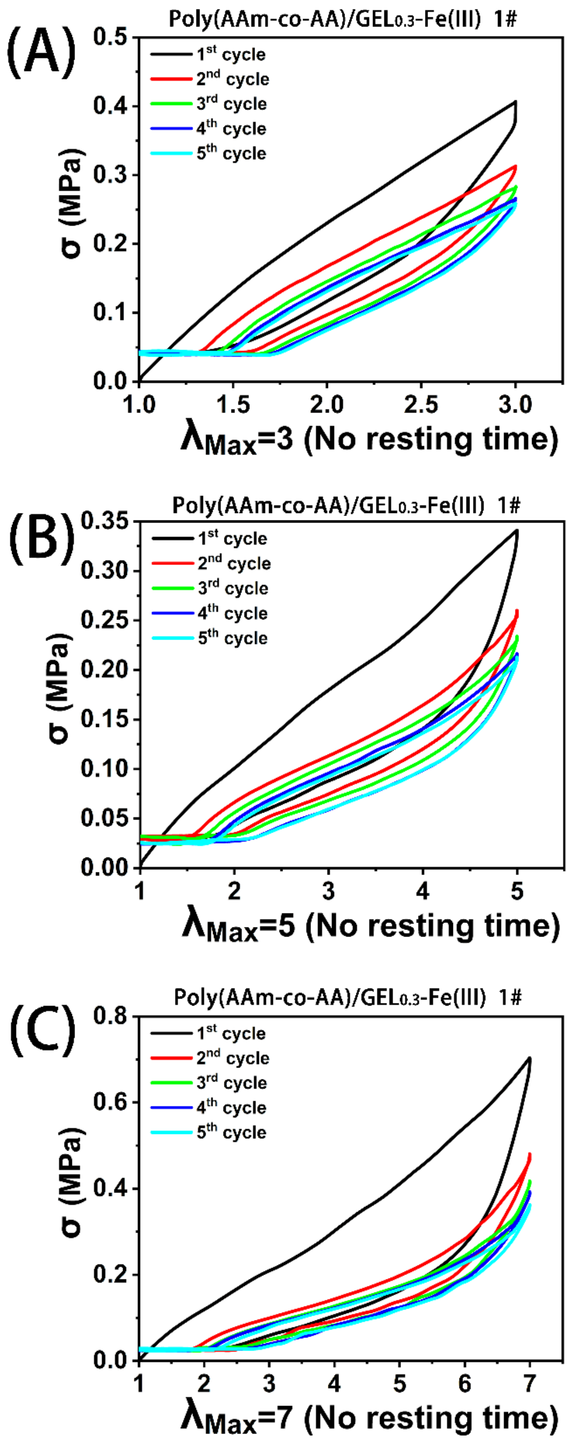

Figure S20 5 consecutive loading-unloading tests of sample 1# with no resting time between two successive tests. (A), (B), and (C) are corresponded to λ_{max} of 3, 5, and 7, respectively.

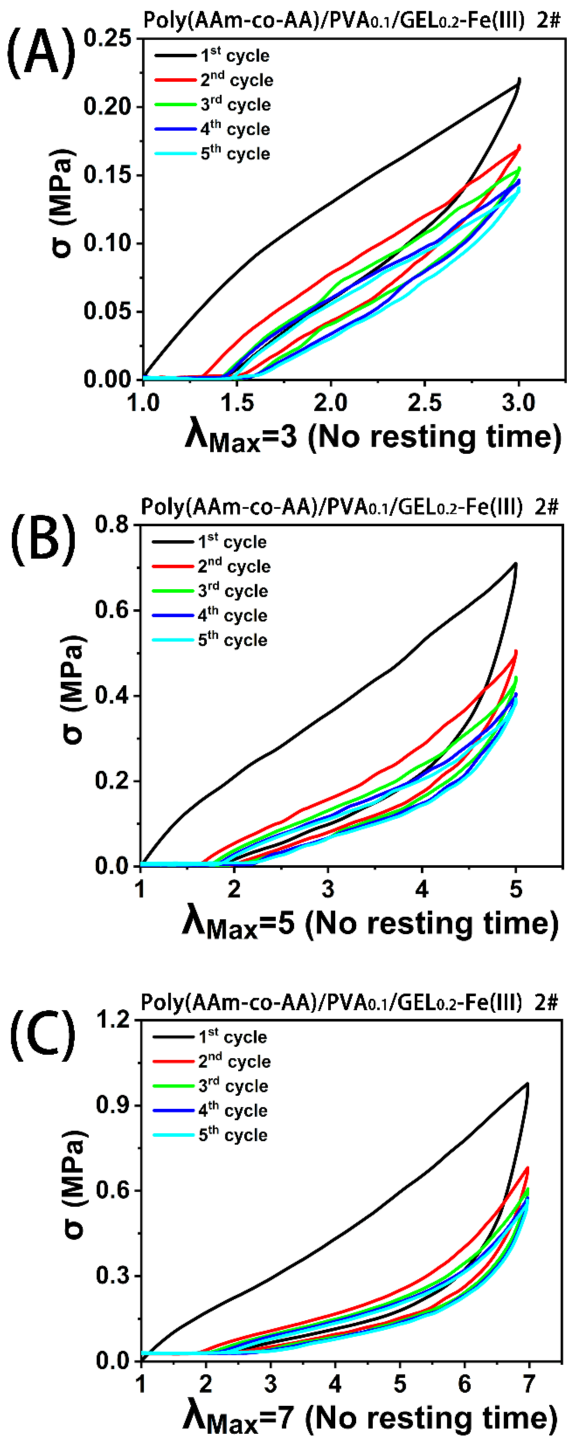

Figure S21 5 consecutive loading-unloading tests of sample 2# with no resting time between two successive tests. (A), (B), and (C) are corresponded to λ_{max} of 3, 5, and 7, respectively.

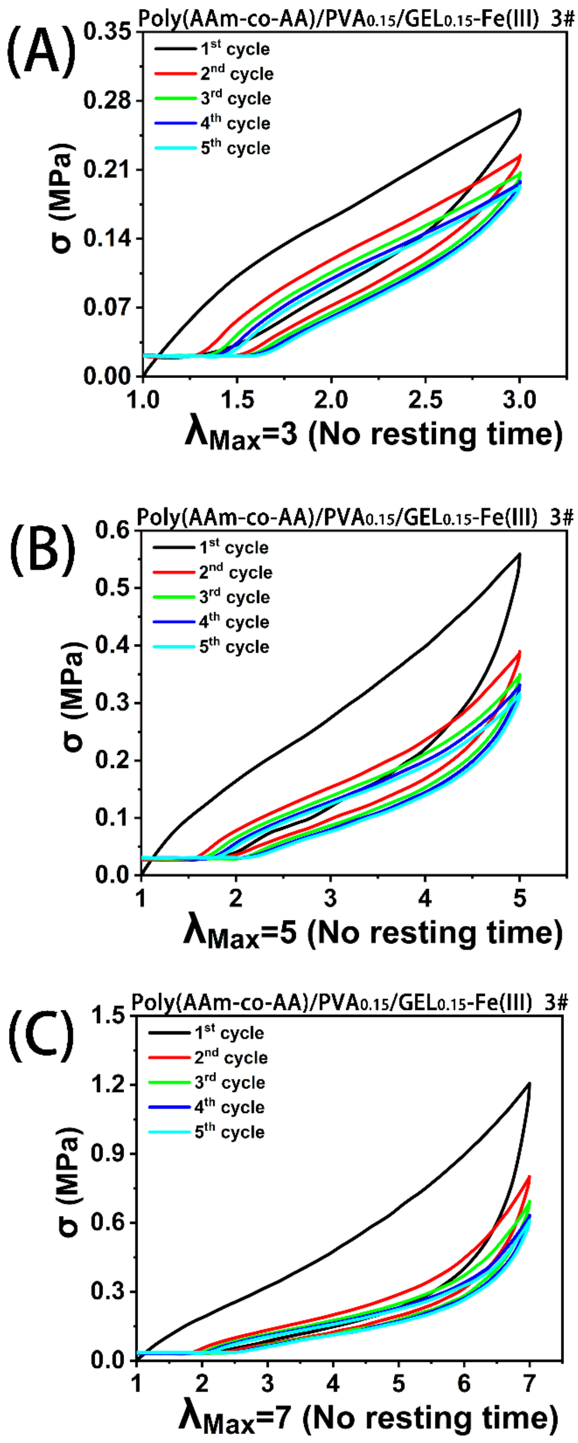

Figure S22 5 consecutive loading-unloading tests of sample 3# with no resting time between two successive tests. (A), (B), and (C) are corresponded to λ_{max} of 3, 5, and 7, respectively.

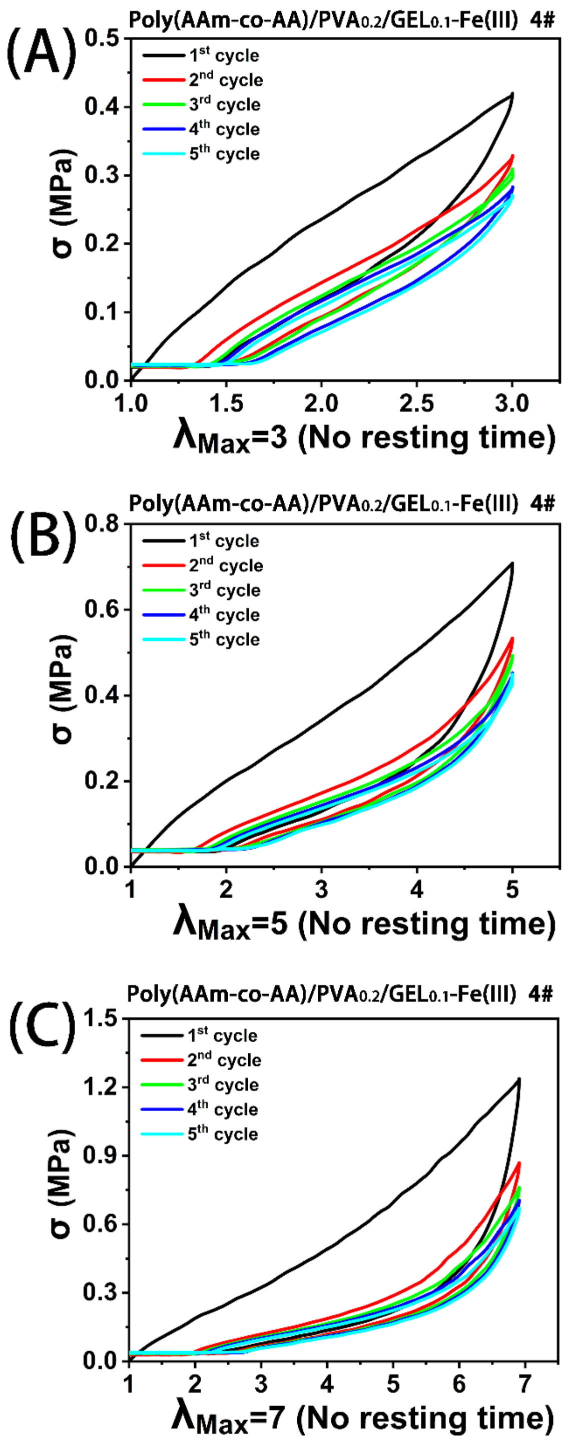

Figure S23 5 consecutive loading-unloading tests of sample 4# with no resting time between two successive tests. (A), (B), and (C) are corresponded to λ_{max} of 3, 5, and 7, respectively.

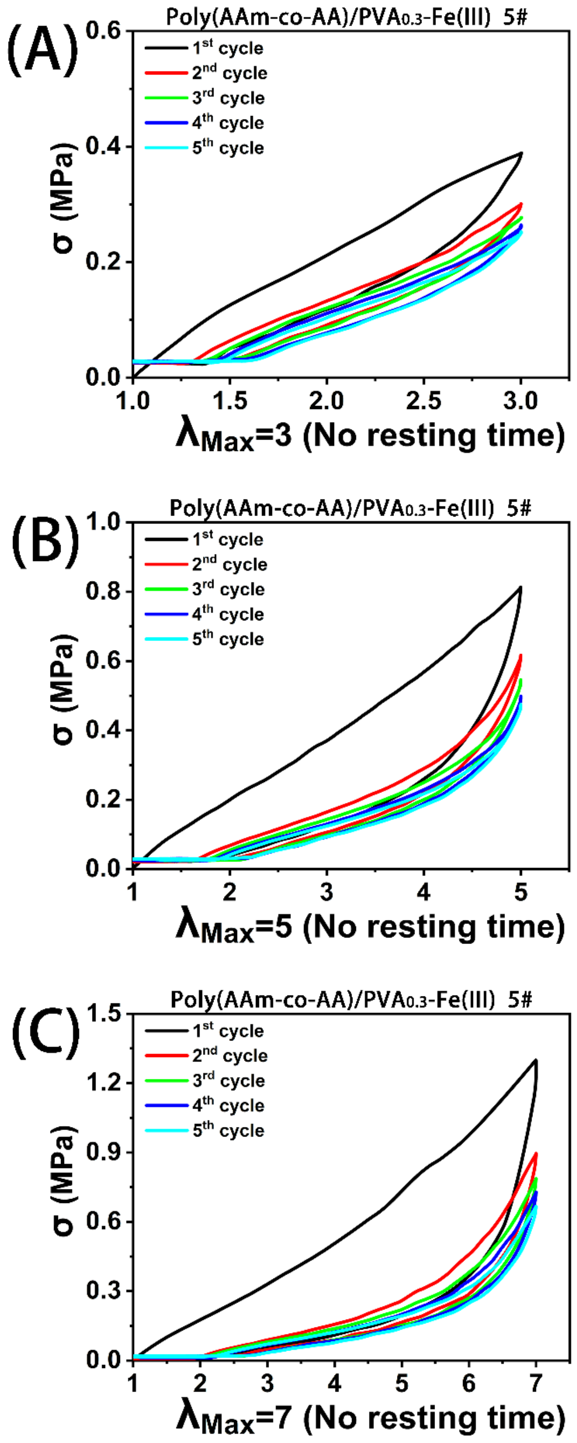

Figure S24 5 consecutive loading-unloading tests of sample 5# with no resting time between two successive tests. (A), (B), and (C) are corresponded to λ_{max} of 3, 5, and 7, respectively.

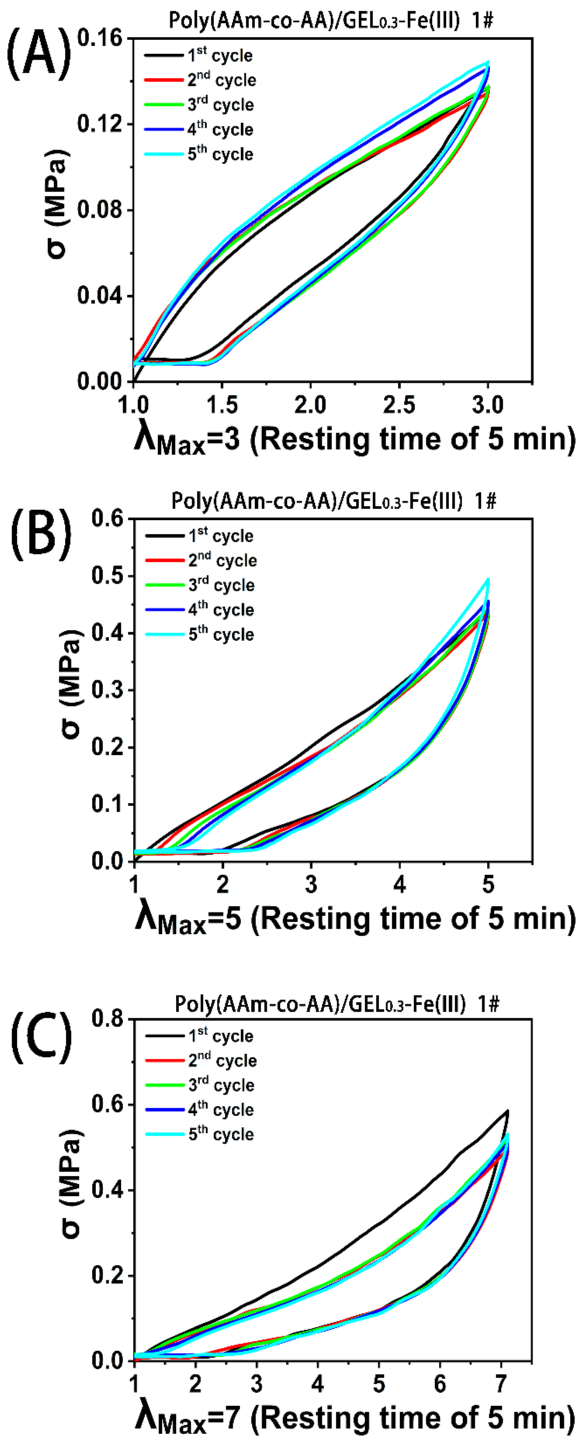

Figure S25 5 consecutive loading-unloading tests of sample 1# with 5 min' resting time between two successive tests. (A), (B), and (C) are corresponded to λ_{max} of 3, 5, and 7, respectively.

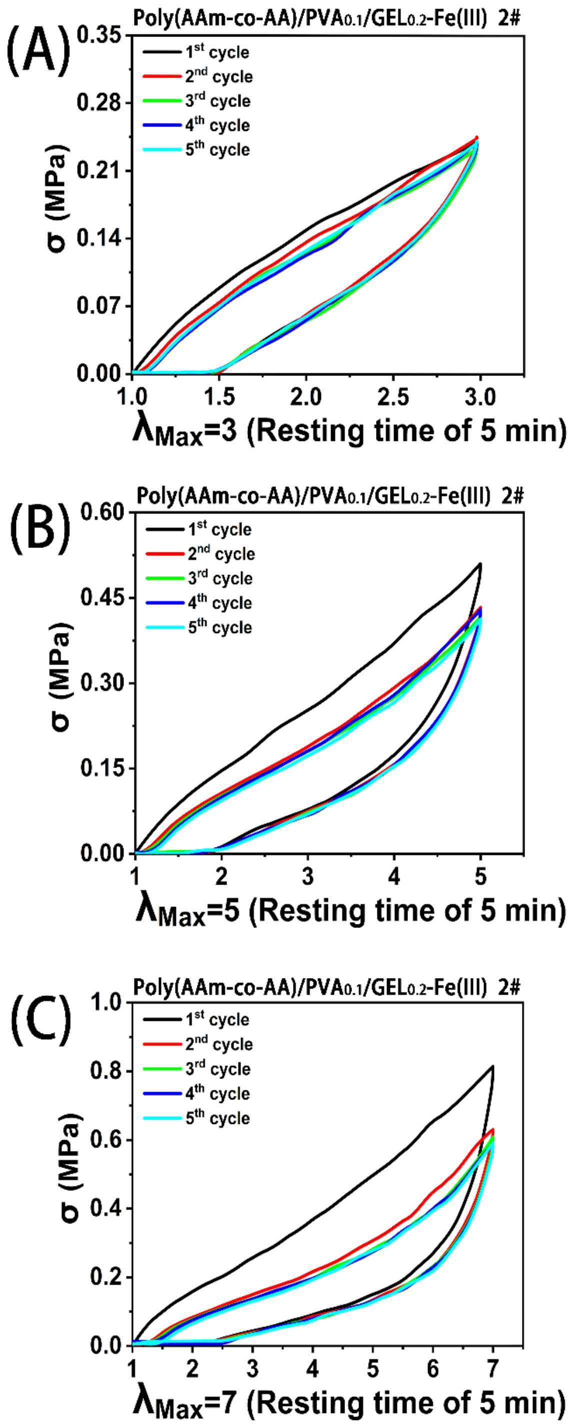

Figure S26 5 consecutive loading-unloading tests of sample 2# with 5 min' resting time between two successive tests. (A), (B), and (C) are corresponded to λ_{max} of 3, 5, and 7, respectively.

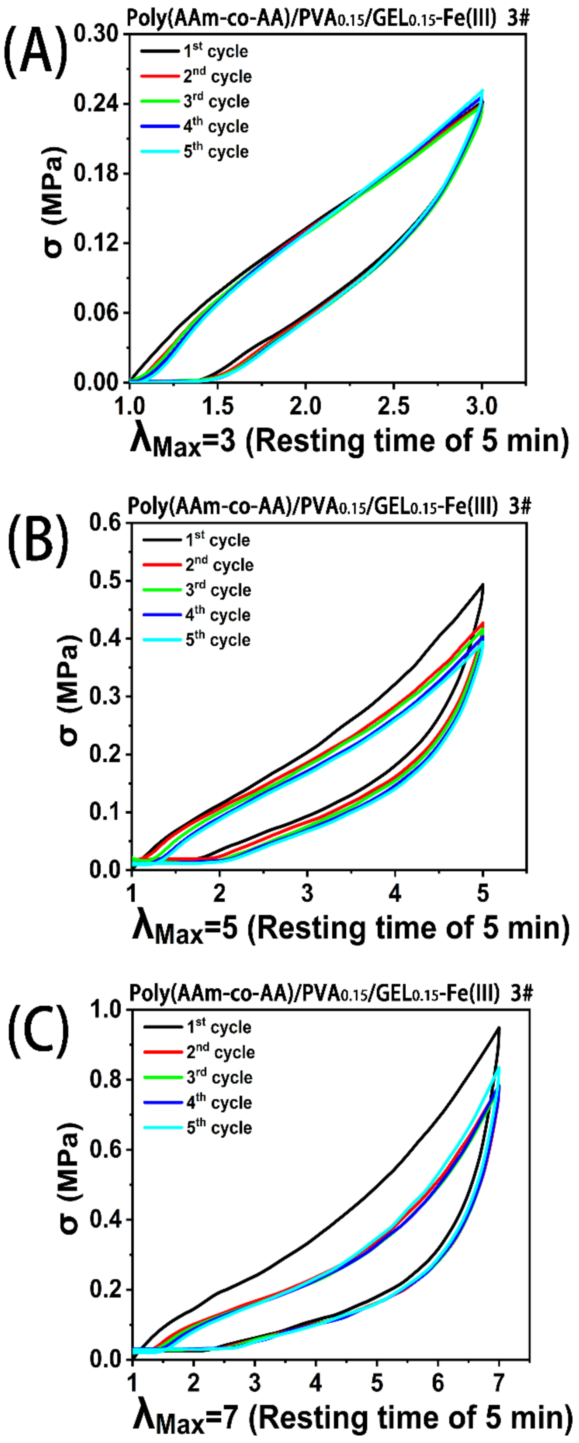

Figure S27 5 consecutive loading-unloading tests of sample 3# with 5 min' resting time between two successive tests. (A), (B), and (C) are corresponded to λ_{max} of 3, 5, and 7, respectively.

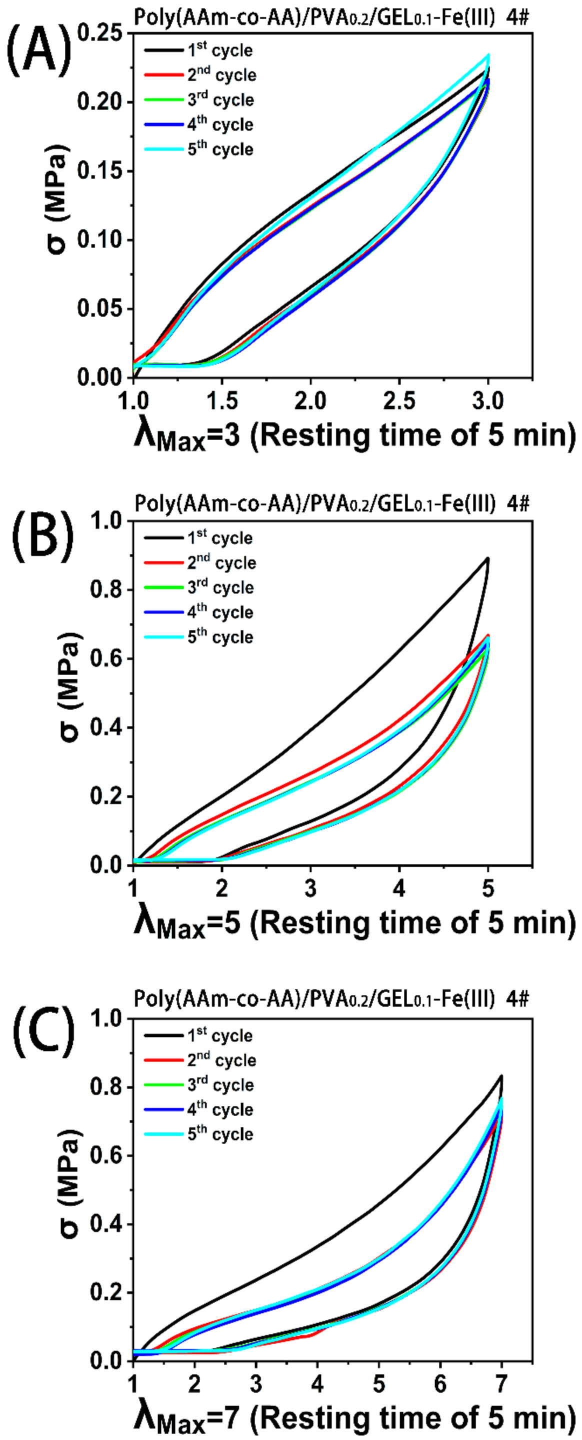

Figure S28 5 consecutive loading-unloading tests of sample 4# with 5 min' resting time between two successive tests. (A), (B), and (C) are corresponded to λ_{max} of 3, 5, and 7, respectively.

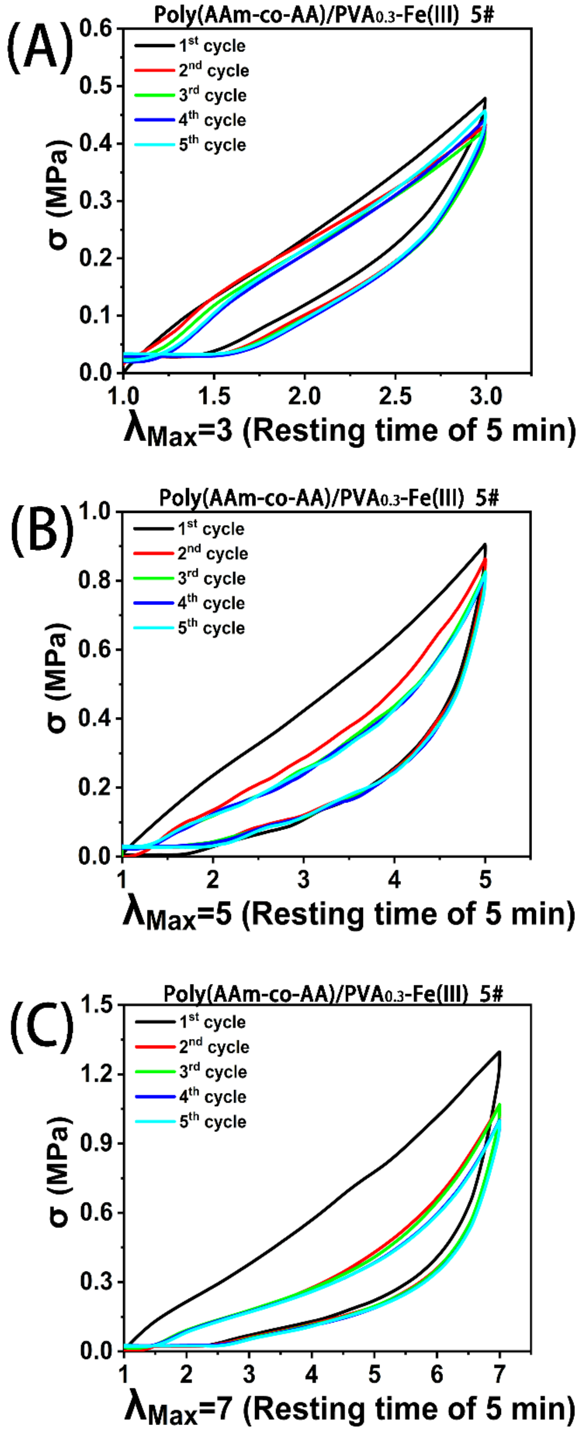

Figure S29 5 consecutive loading-unloading tests of sample 5# with 5 min' resting time between two successive tests. (A), (B), and (C) are corresponded to λ_{max} of 3, 5, and 7, respectively.

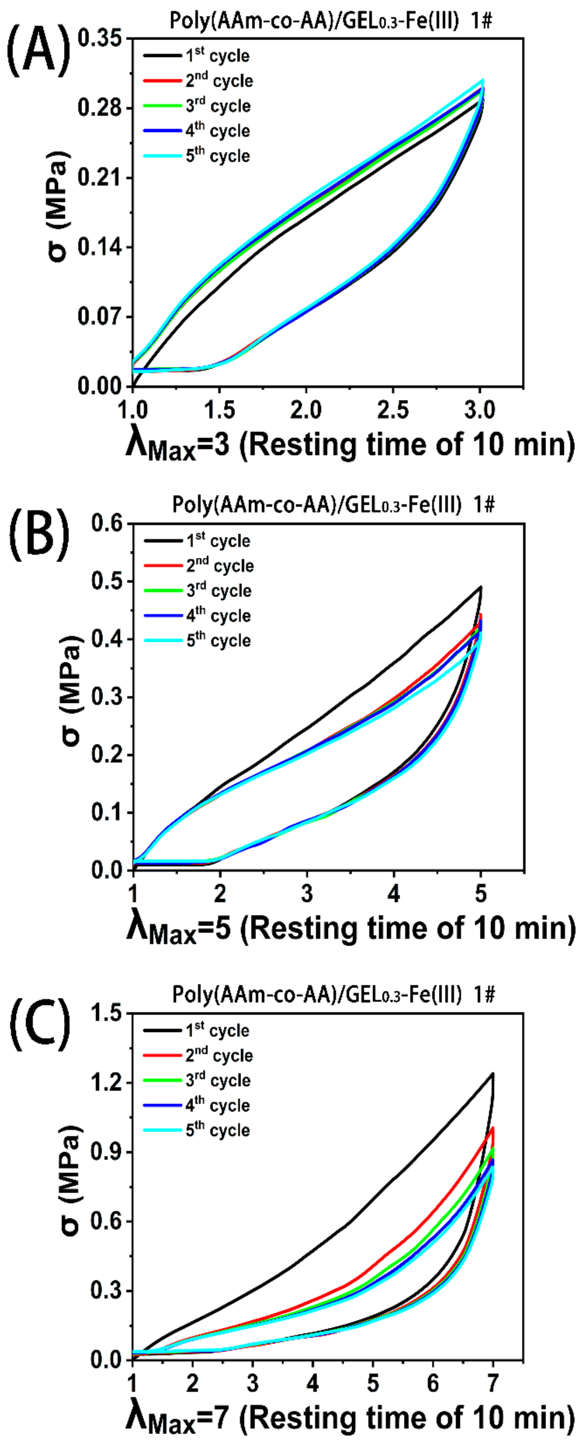

Figure S30 5 consecutive loading-unloading tests of sample 1# with 10 min' resting time between two successive tests. (A), (B), and (C) are corresponded to λ_{max} of 3, 5, and 7, respectively.

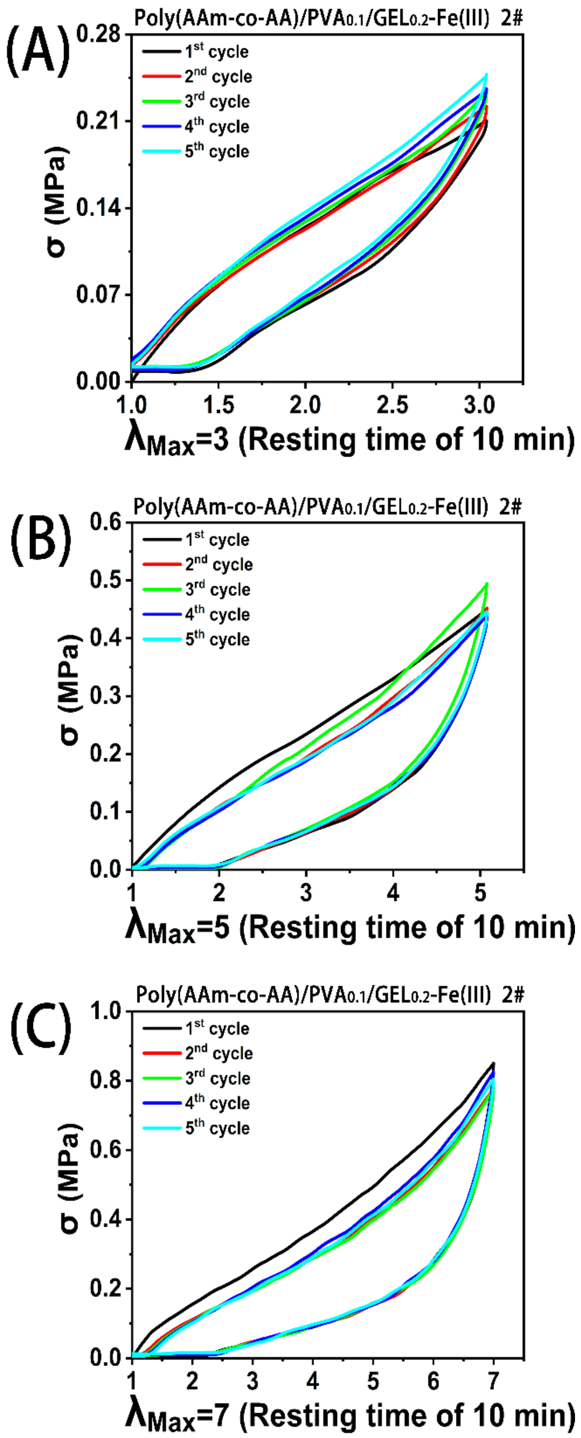

Figure S31 5 consecutive loading-unloading tests of sample 2# with 10 min' resting time between two successive tests. (A), (B), and (C) are corresponded to λ_{max} of 3, 5, and 7, respectively.

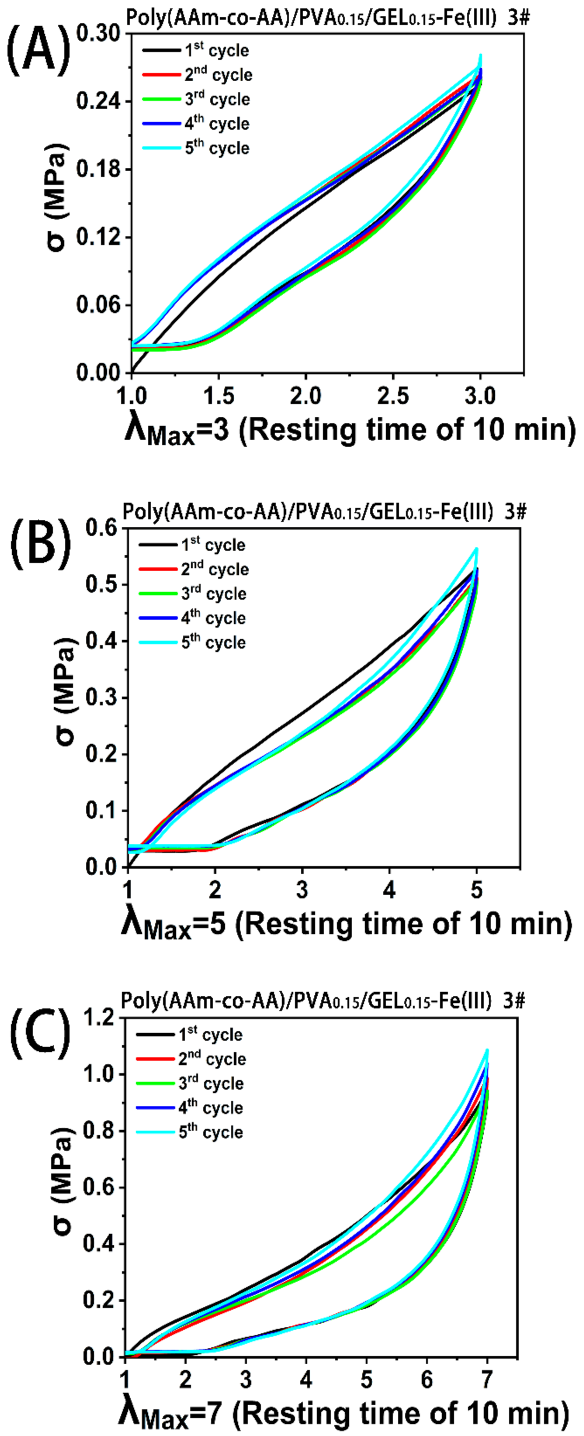

Figure S32 5 consecutive loading-unloading tests of sample 3# with 10 min' resting time between two successive tests. (A), (B), and (C) are corresponded to λ_{max} of 3, 5, and 7, respectively.

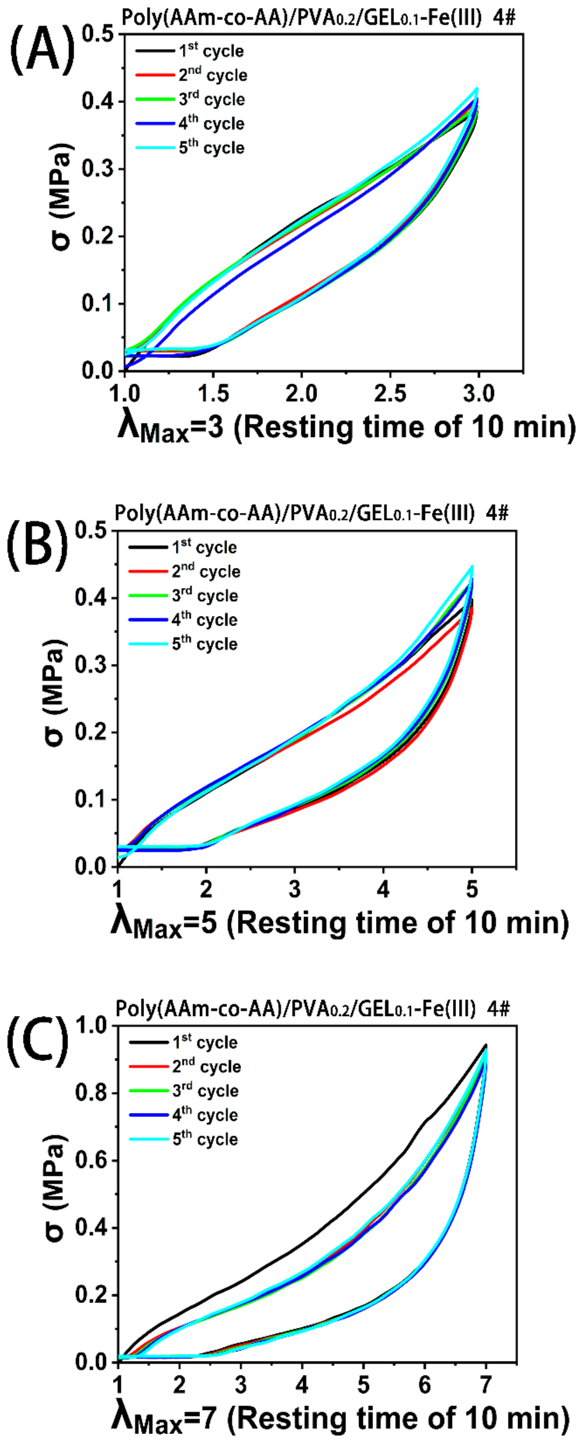

Figure S33 5 consecutive loading-unloading tests of sample 4# with 10 min' resting time between two successive tests. (A), (B), and (C) are corresponded to λ_{max} of 3, 5, and 7, respectively.

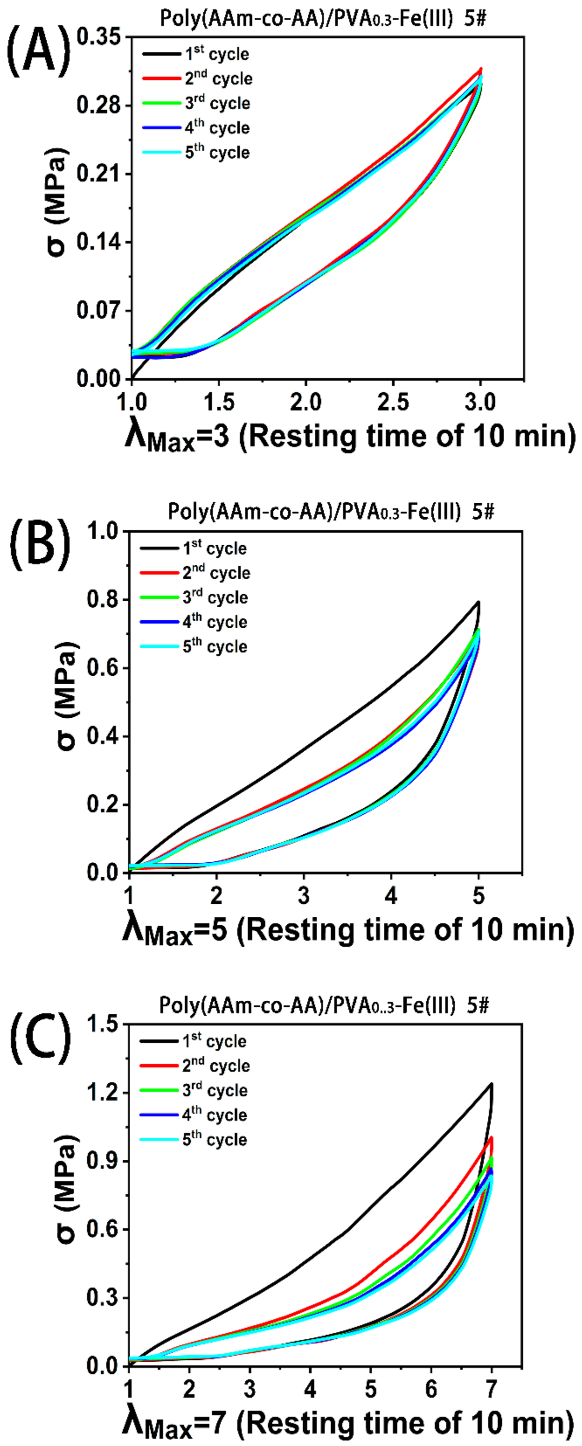

Figure S34 5 consecutive loading-unloading tests of sample 5# with 10 min' resting time between two successive tests. (A), (B), and (C) are corresponded to λ_{max} of 3, 5, and 7, respectively.

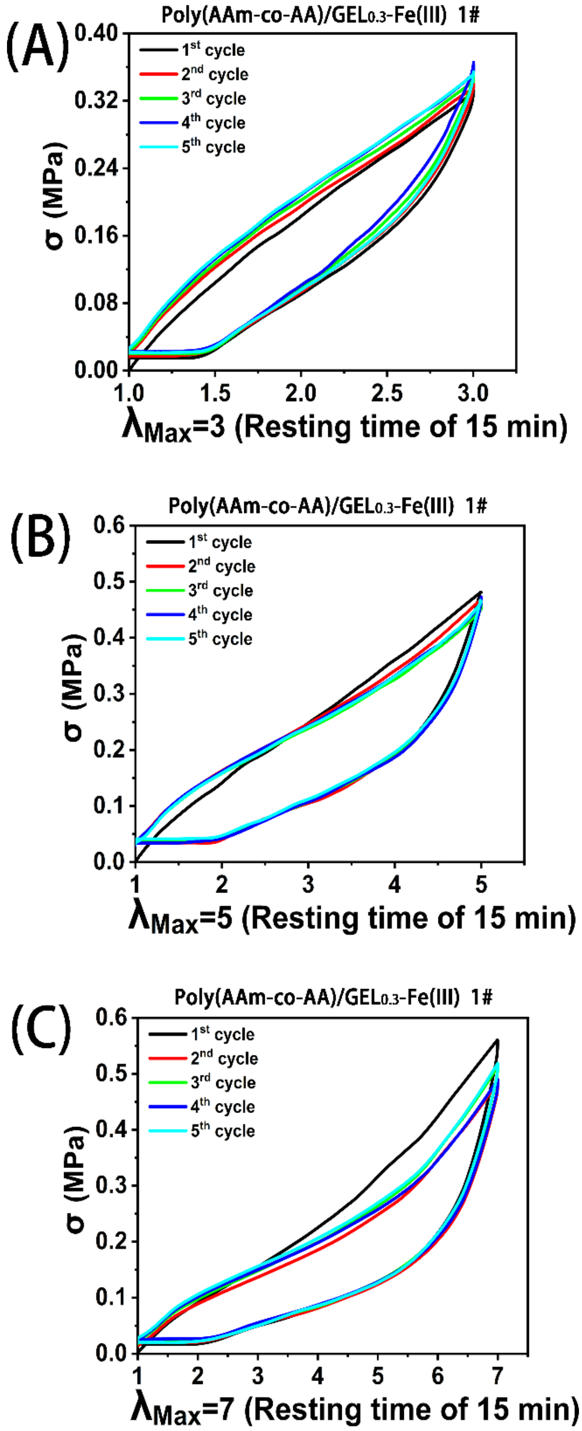

Figure S35 5 consecutive loading-unloading tests of sample 1# with 15 min' resting time between two successive tests. (A), (B), and (C) are corresponded to λ_{max} of 3, 5, and 7, respectively.

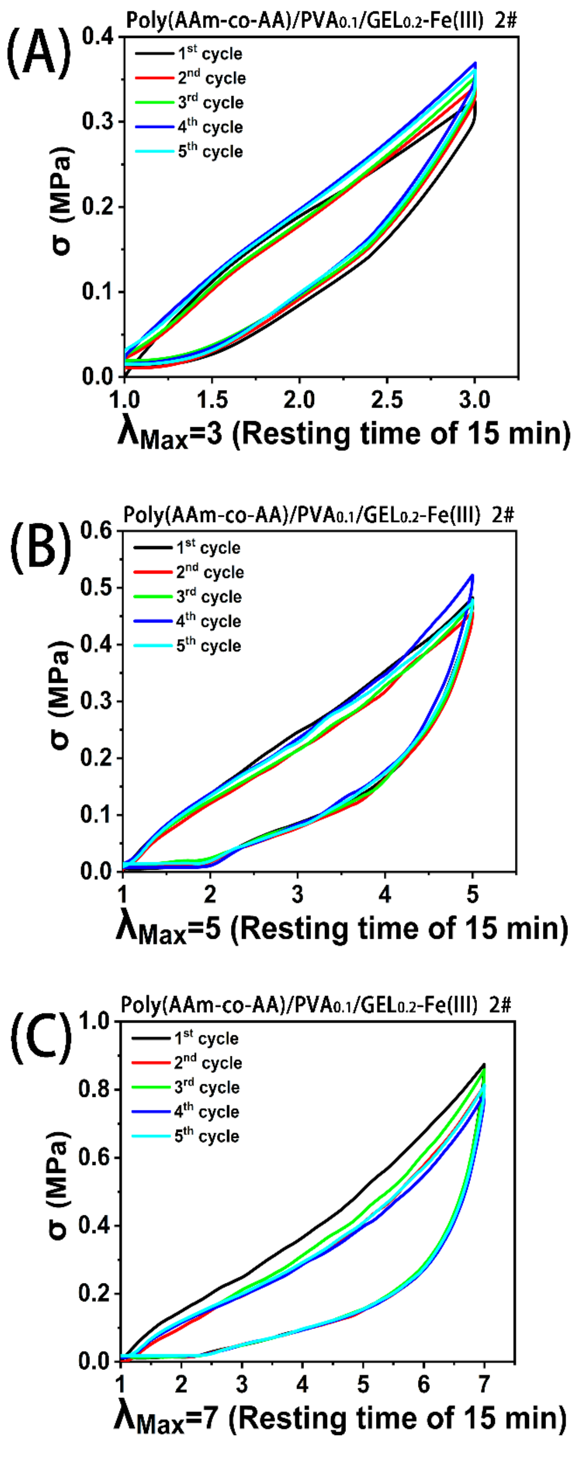

Figure S36 5 consecutive loading-unloading tests of sample 2# with 15 min' resting time between two successive tests. (A), (B), and (C) are corresponded to λ_{max} of 3, 5, and 7, respectively.

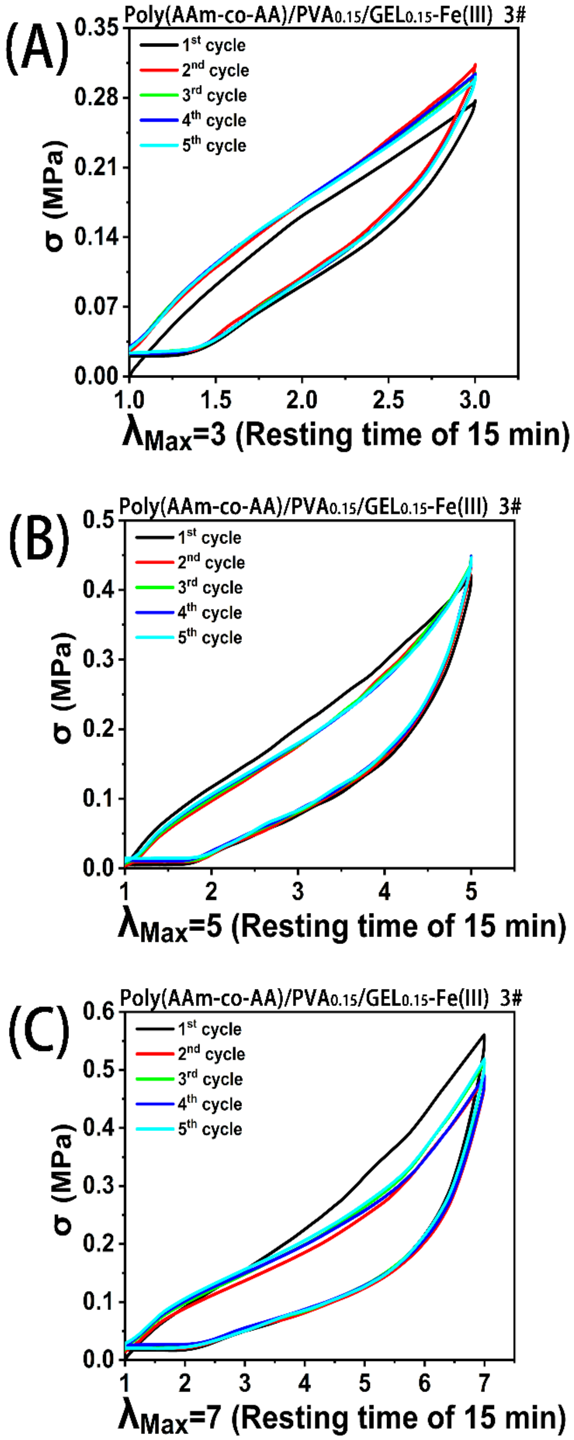

Figure S37 5 consecutive loading-unloading tests of sample 3# with 15 min' resting time between two successive tests. (A), (B), and (C) are corresponded to λ_{max} of 3, 5, and 7, respectively.

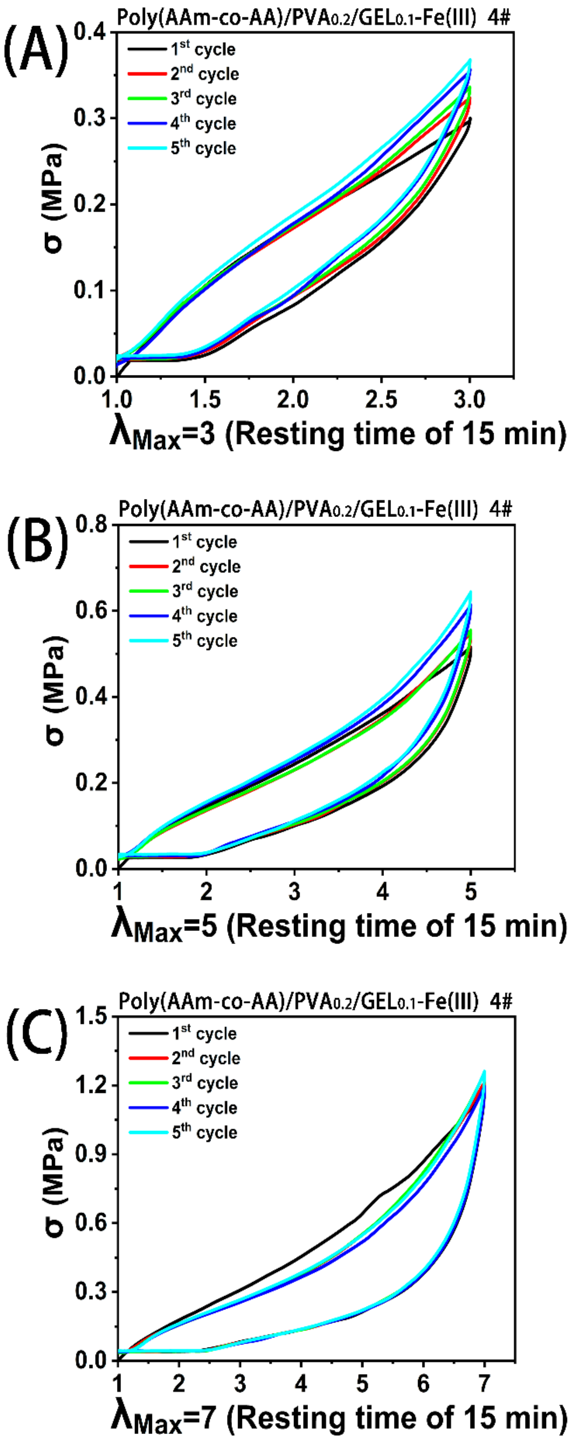

Figure S38 5 consecutive loading-unloading tests of sample 4# with 15 min' resting time between two successive tests. (A), (B), and (C) are corresponded to λ_{max} of 3, 5, and 7, respectively.

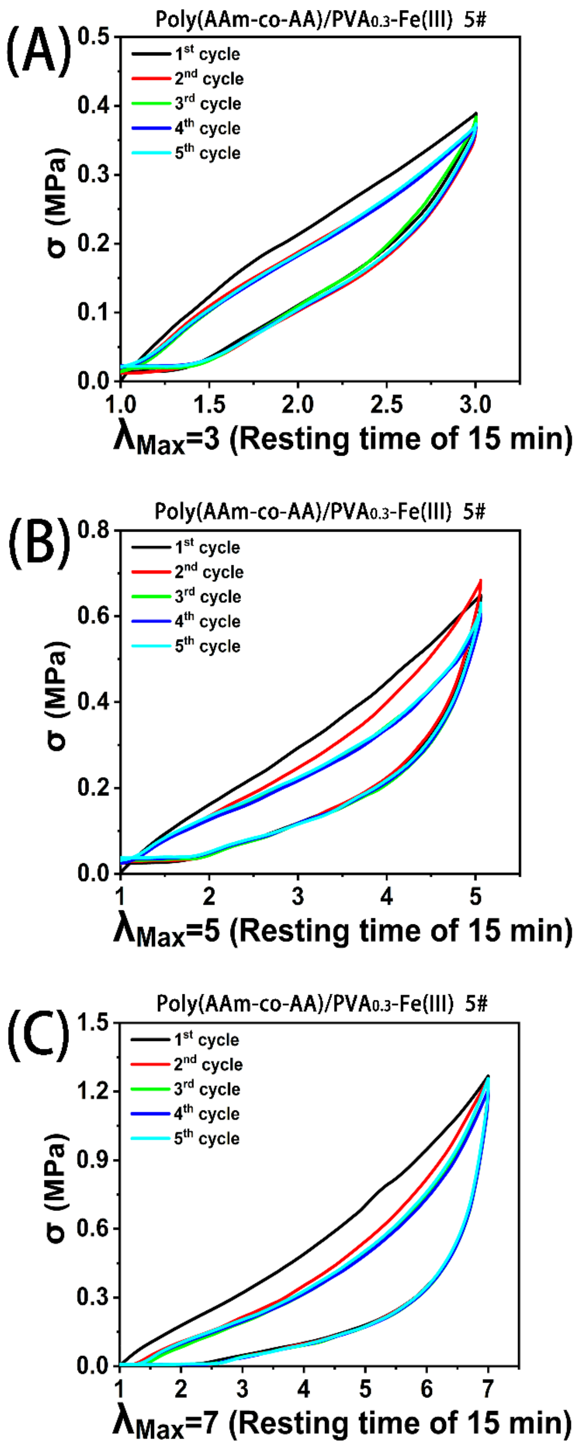

Figure S39 5 consecutive loading-unloading tests of sample 5# with 15 min' resting time between two successive tests. (A), (B), and (C) are corresponded to λ_{max} of 3, 5, and 7, respectively.

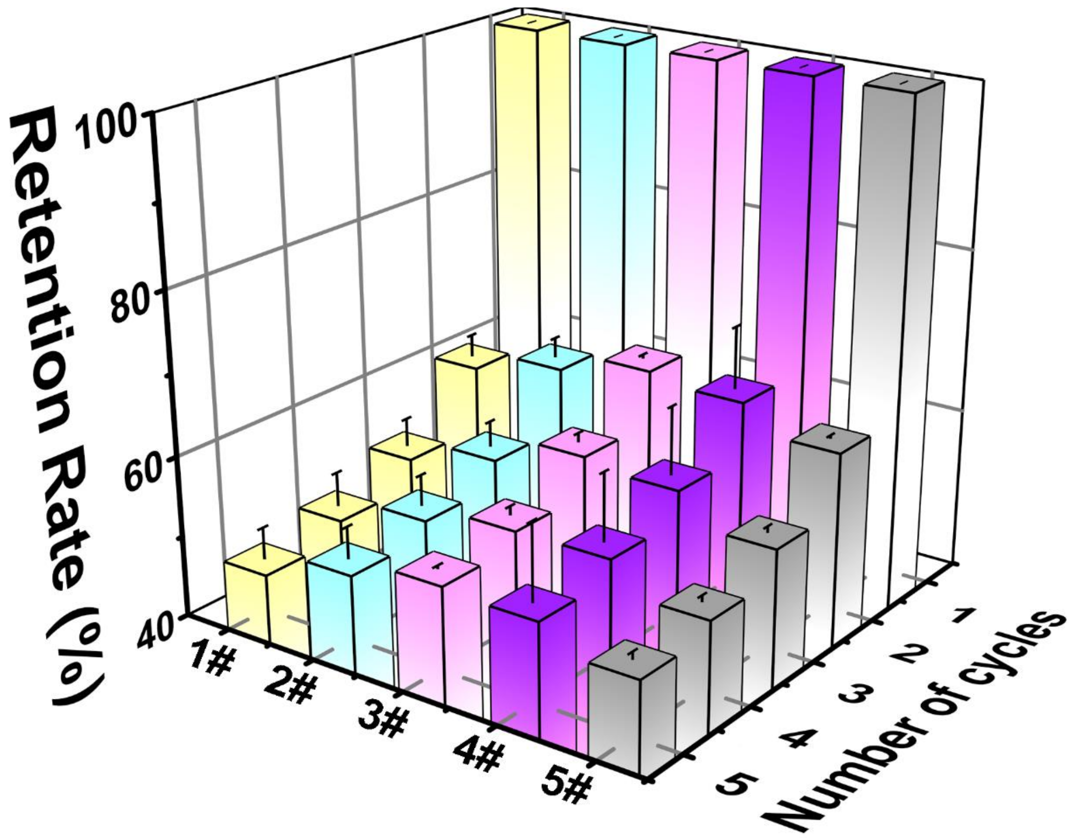

Figure S40 3D histogram summary showing the anti-fatigue capability of sample1#–5# (λ_{max}=5, No resting time)

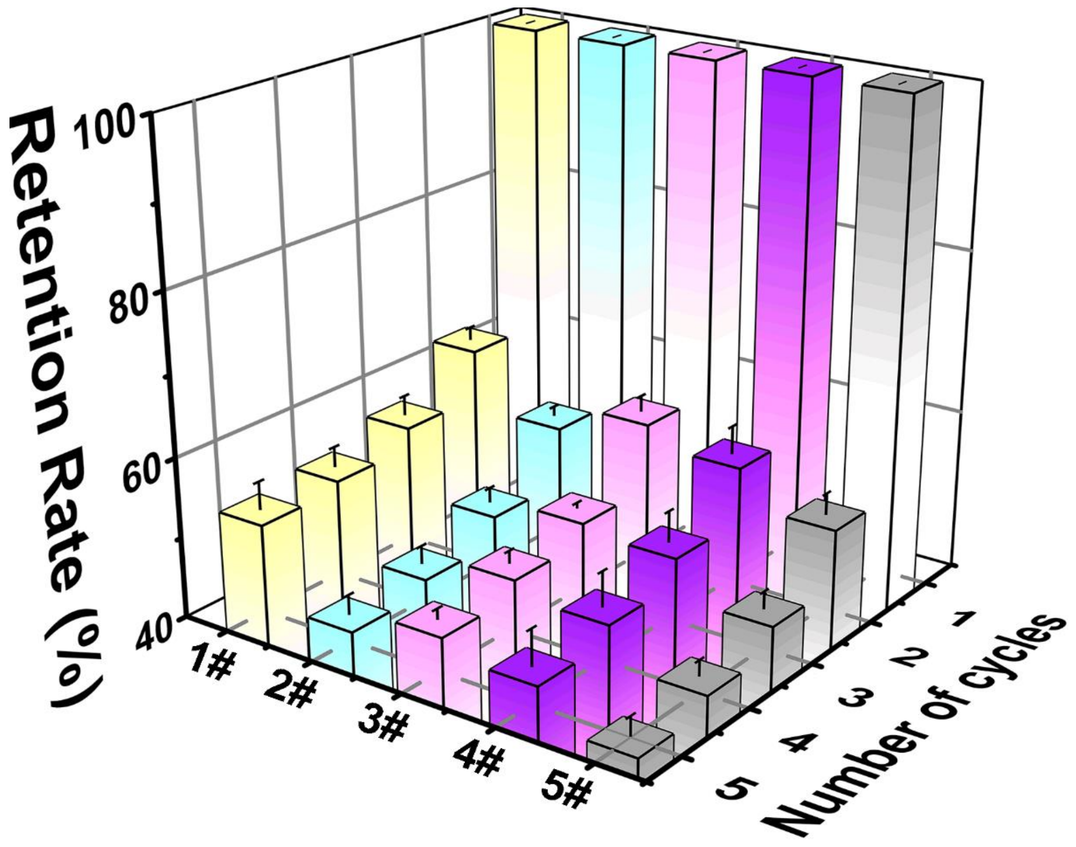

Figure S41 3D histogram summary showing the anti-fatigue capability of sample1#–5# (λ_{max}=7, No resting time).

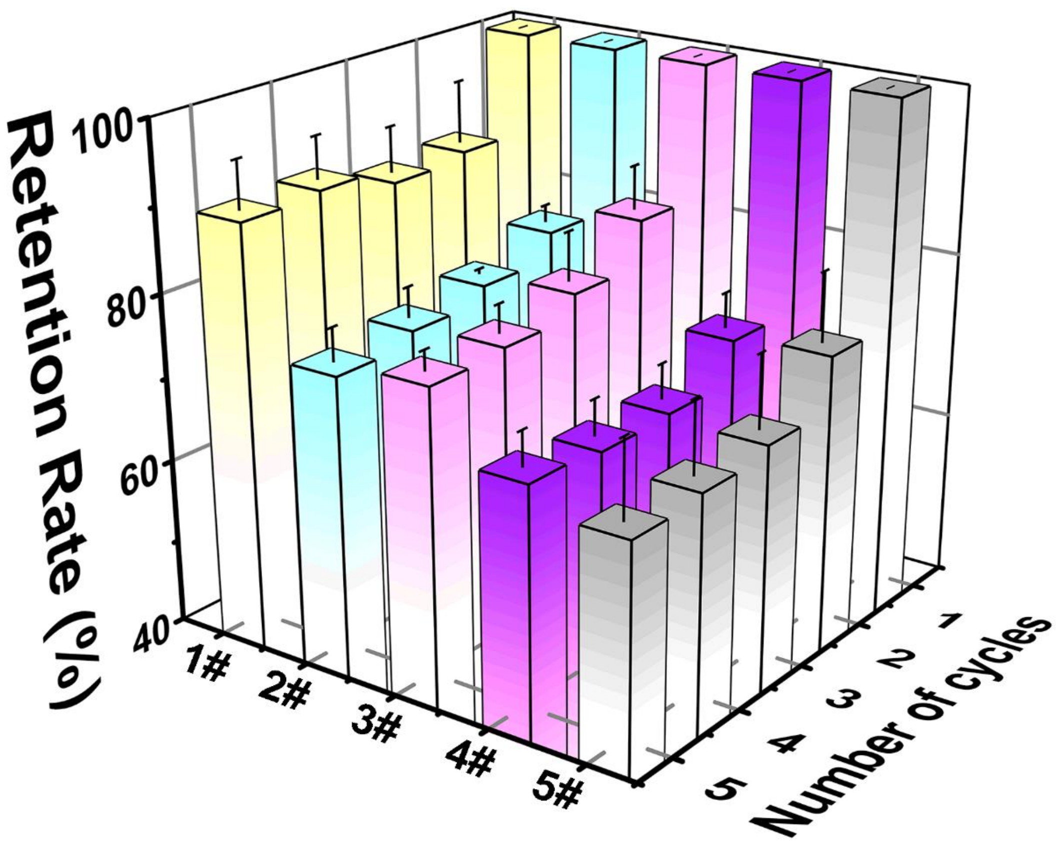

Figure S42 3D histogram summary showing the anti-fatigue capability of sample1#–5# (λ_{max}=5, resting time of 5 min)

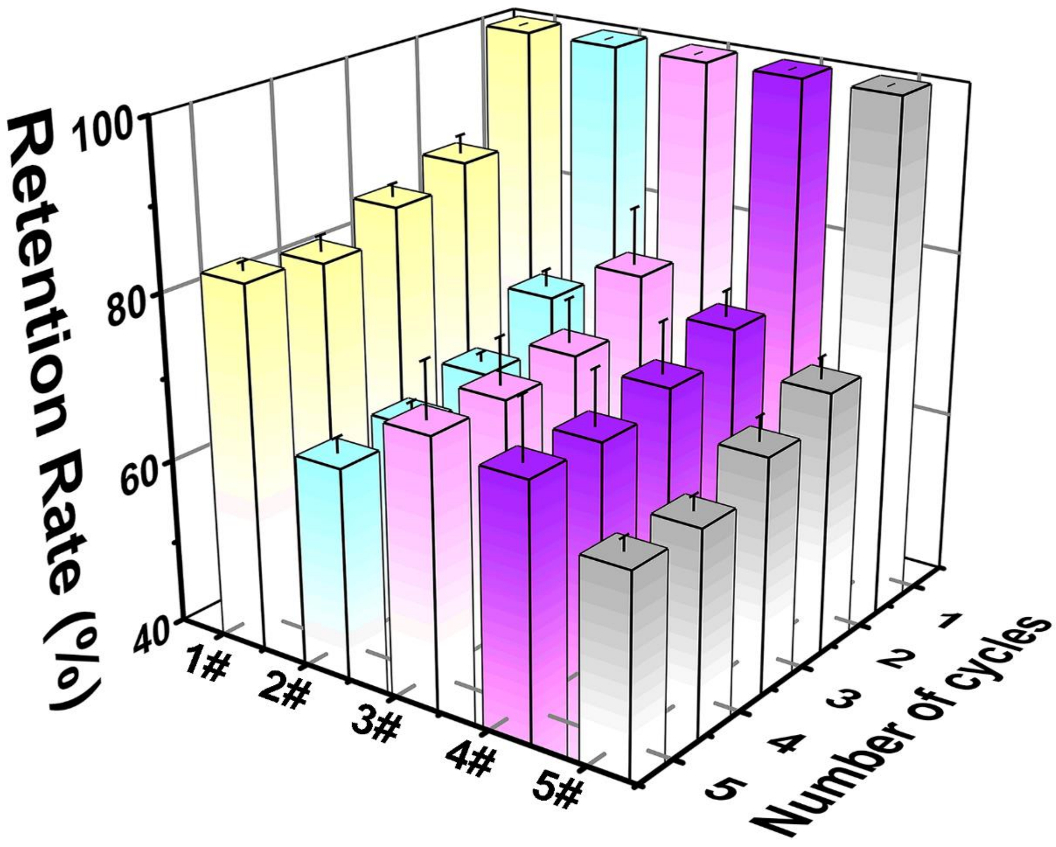

Figure S43 3D histogram summary showing the anti-fatigue capability of sample1#–5# (λ_{max}=5, resting time of 5 min)

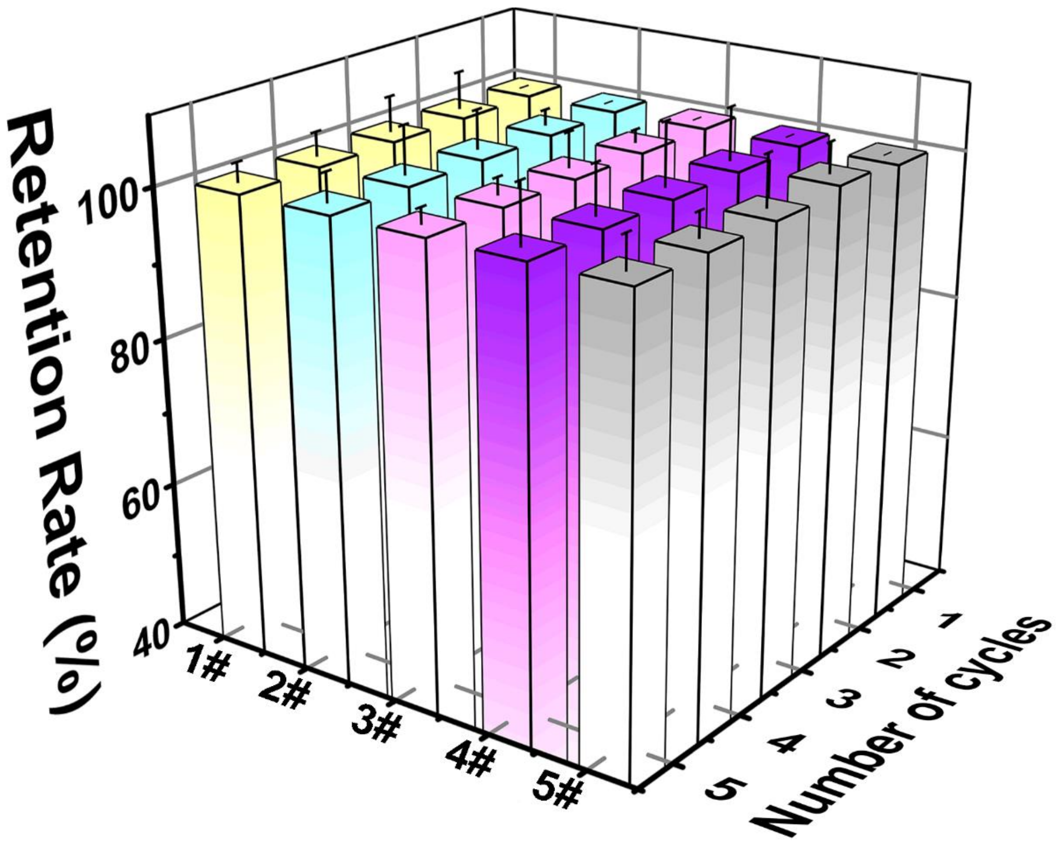

Figure S44 3D histogram summary showing the anti-fatigue capability of sample1#–5# (λ_{max}=3, resting time of 10 min)

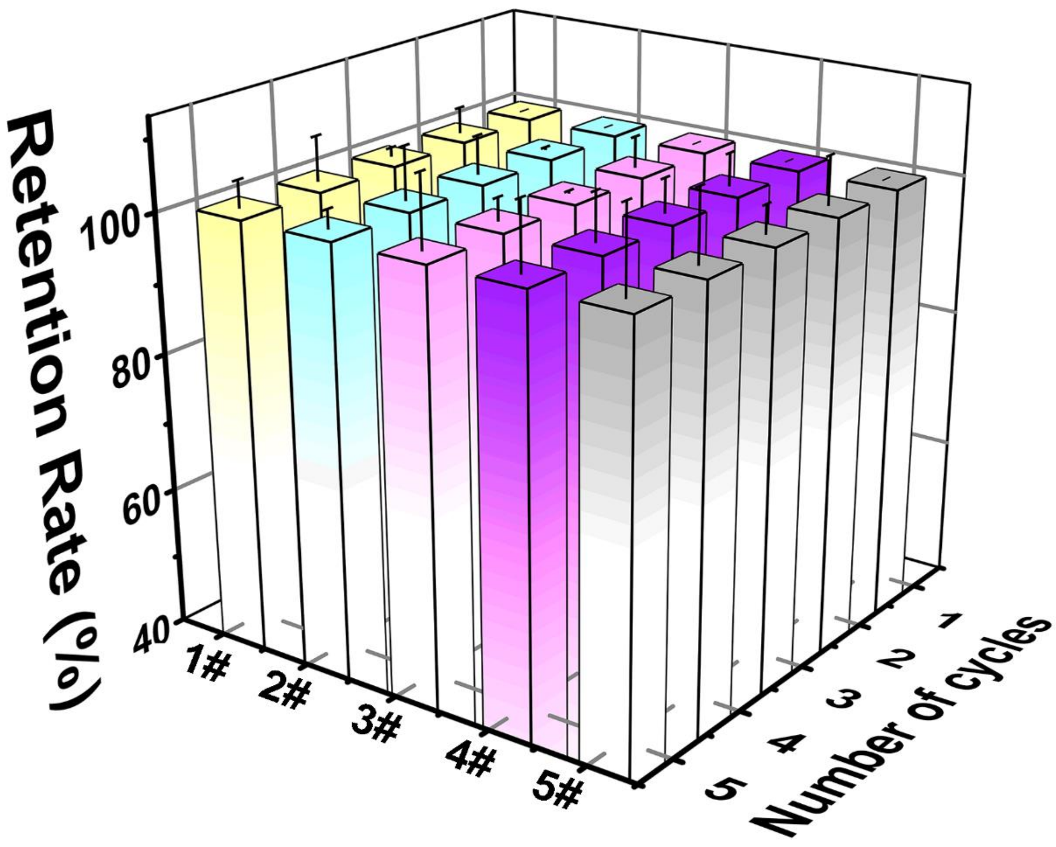

Figure S45 3D histogram summary showing the anti-fatigue capability of sample1#–5# (λ_{max}=3, resting time of 15 min)
